# *Bifidobacterium* spp. and their metabolite lactate protect against acute pancreatitis via inhibition of pancreatic and systemic inflammatory responses

**DOI:** 10.1080/19490976.2022.2127456

**Published:** 2022-10-04

**Authors:** Han Li, Jinyan Xie, Xiuliu Guo, Guilian Yang, Bin Cai, Jingtianyi Liu, Mengjia Yue, Yixin Tang, Gan Wang, Shuxian Chen, Jialin Guo, Xuchen Qi, Donghai Wang, Huijun Zheng, Wei Liu, Hong Yu, Chunfeng Wang, Shu Jeffrey Zhu, Feng Guo

**Affiliations:** a Key Laboratory of Animal Virology of Ministry of Agriculture, Center for Veterinary Sciences, Zhejiang University, Hangzhou, China; b Department of Critical Care Medicine, Sir Run Run Shaw Hospital, Zhejiang University School of Medicine, Hangzhou, China; c College of Veterinary Medicine, Jilin Provincial Engineering Research Center of Animal Probiotics, Jilin Provincial Key Laboratory of Animal Microecology and Healthy Breeding, Jilin Agricultural University, Changchun, China; d Department of Quality Management, Sir Run Run Shaw Hospital, Zhejiang University School of Medicine, Hangzhou, China; e Central Laboratory of Medicine, Shaoxing People’s Hospital, Shaoxing, China; f Institute of Land and Food Systems, University of British Columbia, Vancouver, BC, Canada; g Department of Neurosurgery, Sir Run Run Shaw Hospital, Zhejiang University School of Medicine, Hangzhou, China; h State Key Laboratory of Pathogen and Biosecurity, Beijing Institute of Microbiology and Epidemiology, Beijing, China; i Department of General Surgery, Sir Run Run Shaw Hospital, Zhejiang University School of Medicine, Hangzhou, China

**Keywords:** *Bifidobacterium*, microbial metabolite, lactate, macrophages, pancreatic and systemic inflammation, immunomodulation

## Abstract

Severe acute pancreatitis (SAP) is a critical illness characterized by a severe systemic inflammatory response resulting in persistent multiple organ failure and sepsis. The intestinal microbiome is increasingly appreciated to play a crucial role in modulation of AP disease outcome, but limited information is available about the identity and mechanism of action for specific commensal bacteria involved in AP-associated inflammation. Here we show that *Bifidobacteria*, particularly *B. animalis*, can protect against AP by regulating pancreatic and systemic inflammation in germ-free (GF) and oral antibiotic-treated (Abx) mouse models. Colonization by *B. animalis* and administration of its metabolite lactate protected Abx and GF mice from AP by reducing serum amylase concentration, ameliorating pancreatic lesions and improving survival rate after retrograde injection of sodium taurocholate. *B. animalis* relieved macrophage-associated local and systemic inflammation of AP in a TLR4/MyD88- and NLRP3/Caspase1-dependent manner through its metabolite lactate. Supporting our findings from the mouse study, clinical AP patients exhibited a decreased fecal abundance of *Bifidobacteria* that was inversely correlated with the severity of systemic inflammatory responses. These results may shed light on the heterogeneity of clinical outcomes and drive the development of more efficacious therapeutic interventions for AP, and potentially for other inflammatory disorders.

## Introduction

Acute pancreatitis (AP) is an inflammatory disorder of the pancreas that is characterized by abnormal activation of trypsinogen within pancreatic acinar cells, resulting in autodigestion of the pancreas and local or even systemic inflammation.^1^ Although most AP only results in self-limiting mild disease, a subpopulation of patients progresses to severe systemic inflammatory response syndrome (SIRS) and multiple organ dysfunction syndrome (MODS), which can cause critical illness requiring long-term ICU care.^2^ Complications of necrotizing pancreatitis including persistent multiple organ failure (PMOF) and sepsis enormously exacerbate severe acute pancreatitis (SAP), causing mortality at astonishing rates of 30% or even higher.^3^ Moreover, despite the global burden of the disease, no effective therapeutic agents are currently available to treat or prevent AP.

Cumulative data strongly support the view that the systemic complications and severity of AP are greatly determined by the intensity of the immune response.^1,2^ Injured pancreatic acinar cells release proinflammatory cytokines and chemokines that recruit infiltrating mononuclear-phagocytes and neutrophils to the injury site.^3,4^ Subsequently, the cellular contents released from necrotic and injured cells activate monocytes and further propagate pancreatic inflammation, which damages the pancreas and mediates macrophage activation in distant organs, leading to overwhelming SIRS and PMOF in the worst-case scenario.^5,6^ Given their pivotal role in activation and amplification of inflammatory responses in AP, the NF-κB, and inflammasome signaling pathways have been key targets for the development of inhibitors looking to cure the disease over the past decade.^4,7,8^

Cellular innate immunity sensors including Toll-like receptors (TLRs) such as TLR4 and TLR9, and nucleotide-binding oligomerization domain (NOD)-like receptors (NLRs) such as NOD1 are believed to be major contributors to the progression of AP.^9–11^ These sensors recognize damage-associated molecular patterns (DAMPs) or pathogen-associated molecular patterns (PAMPs) as the first signals to induce NF-κB complex formation and activation, which upregulates procytokines of pro-IL-1β and pro-IL18 and inflammasome components.^12^ A secondary signal, provided by DAMPs released from damaged acinar cells, causes the assembly and activation of inflammasome complexes.^11^ Among them, NLR pyrin domain-containing protein 3 (NLRP3) is the most extensively studied inflammasome sensor protein, and it is a strong determinant of the inflammatory responses and pancreatic tissue damage associated with AP.^13^ However, the environmental factors that influence TLR and NLRP3 inflammasome signaling in mononuclear phagocytes and the consequent impact on systemic inflammatory responses in AP remain largely unknown.

Perturbation of the intestinal microbiota plays a role in accelerating the systemic inflammation of AP.^14,15^ Accumulating evidence has shown that AP cases can have increased intestinal permeability, and translocation of the intestinal microbiome is thought to be associated with complications such as abdominal infection and sepsis.^16,17^ AP induced by caerulein administration in antibiotic (Abx)-treated or germ-free (GF) mice exhibited lower pancreatic inflammation and decreased production of systemic proinflammatory cytokines, whereas fecal microbiota transplantation (FMT) worsened the disease, suggesting that the gut commensal bacteria as a whole exacerbates AP.^10,14^ On the other hand, probiotics are a promising protective treatment against SAP, considering that specific probiotic strains under the right conditions can alleviate AP.^18,19^ Unfortunately, a comprehensive understanding of how the intestinal microbiota is linked to inflammatory signaling is lacking, and specific commensal microorganisms that are capable of limiting inflammatory responses in AP are yet to be identified.

In this study, we discovered that the commensal *Bifidobacterium* spp. are able to ameliorate both local and systemic inflammation from AP. Using *B. animalis* as the model organism, we determined that colonization of Abx-treated or GF mice resulted in less severe pancreatic injury and diminished local and systemic inflammatory responses in a TLR4/MyD88- and NLRP3/Caspase1-dependent manner. Exogenous administration of lactate, one of the main metabolites produced by *B. animalis*, to Abx-treated and GF mice recapitulated the reduction of TLR4-MyD88- and NLRP3 inflammasome-mediated inflammation observed with *B. animalis* monocolonization. Thus, we identified a gut-pancreas axis that integrates environmental, metabolic, and immunologic cues to regulate the local and systemic inflammation mediated by NF-κB and NLRP3 inflammasome pathways in AP.

## Results

### Intensive short-term antibiotics treatment via oral gavage exacerbates caerulein-induced acute pancreatitis

**Figure 1.**
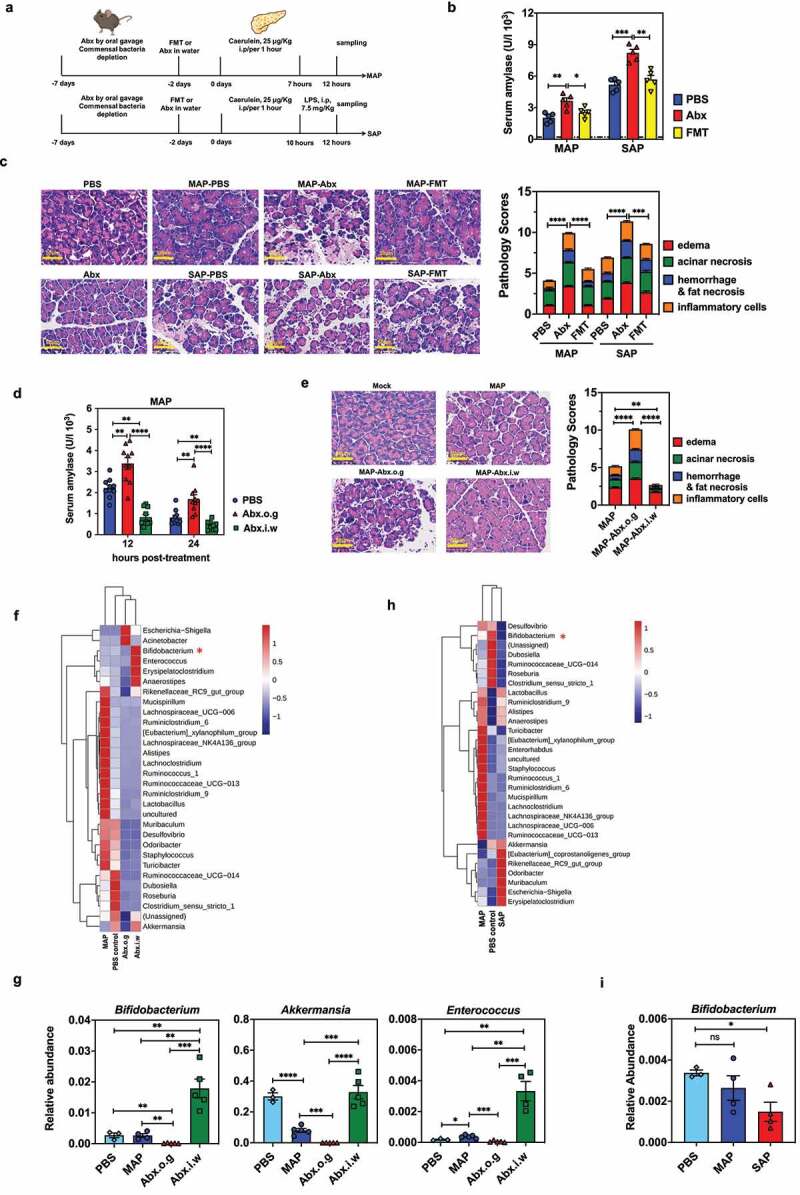
Intensive short-term antibiotic treatment via oral gavage exacerbates caerulein-induced acute pancreatitis. (a) Schematic illustration of experiments in MAP and SAP models induced by caerulein injection, including PBS, antibiotic (Abx) treatment and fecal microbiota transplantation (FMT). (b) Serum amylase levels and (c) representative H&E staining of pancreatic sections with pathology scores for SAP or MAP mice at 12 h post-treatment (hpt) with PBS, Abx or FMT (n = 5 each). (d) Serum amylase activity of MAP mice treated with PBS, Abx oral gavage (o.g.) or Abx in drinking water (i.w.) at 12 or 24 hpt (n = 10 each). (e) Pathological sections and pancreatic pathology scores from mice from (d) at 24 hpt. (f) The microbiota composition in fecal samples collected from groups treated with PBS, Abx o.g. or Abx i.w. was analyzed at the genus level by 16S rDNA sequencing (n = 3–5). (g) The relative abundance of *Bifidobacterium, Akkermansia* and *Enterocococcus* in fecal samples collected from (f). (h) 16S rDNA sequencing analysis from fecal samples of PBS control, MAP and SAP mice (n = 3–5). (i) Relative abundance of *Bifidobacterium* from (h). Data are from two independent experiments, and *P* values were determined by unpaired two-tailed Student’s t-test; *: *P* < .05; **: *P* < .01; ***: *P* < .001; ****: *P* < .0001.

Recent studies demonstrated reduced pancreatic inflammation in Abx-treated or GF mice in a caerulein-induced model of AP, implicating the intestinal microbiota in disease progression.^10,14^ Thus, we logically predicted that pancreatic inflammation would be attenuated under conditions of microbiota depletion. We used a more intensive 5-day regimen of highly concentrated Abx by oral gavage, as described previously.^20^ MAP or SAP was then induced in these animals by caerulein injection (Figure 1a). Surprisingly, mice with orally administered Abx exhibited more severe pancreatic inflammatory lesions and a markedly higher level of serum amylase than PBS-treated controls in both MAP and SAP models. FMT from PBS control mice into Abx-treated mice reversed the amylase and lipase concentration differences in the serum (Figure 1b and Fig. S1A) and prevented pancreatic inflammation (Figure 1c), suggesting that these phenotypes were due to intestinal microbiome alteration. There was no discrepancy in the baseline of serum amylase or pancreatic histology between PBS- and Abx-treated, caerulein-untreated mice (Figure 1c and Fig. S1B). Fecal 16S rDNA copy numbers suggested that FMT reconstituted the enteric bacterial abundance before AP induction (Fig. S1C).

Because our results were contradictory to the study conducted by Tsuji et al.,^10^ we hypothesized that the distinct phenotype regarding resistance to AP induction could be attributed to the utilization of a different Abx treatment regimen. To confirm this hypothesis, in parallel, we depleted the intestinal microbiota either with a short-term (5-day) oral gavage, or with a longer-term Abx administration (3 weeks) in the drinking water as done in the Tsuji study. Indeed, mice given Abx in the drinking water showed barely detectable pancreatic inflammation and only mild elevation of serum amylase, whereas mice receiving 5-day oral gavage with a more concentrated Abx cocktail developed more severe pancreatic pathology and drastically increased serum amylase concentration compared with PBS control animals (Figure 1d,e).

Based on these observations, we speculated that mice with long-term Abx pretreatment in the drinking water had acquired bacterial communities that conferred resistance to AP, while these critical commensal bacteria had very likely been eliminated in our mouse model after short-term intensive Abx oral gavage. Thus, we analyzed the changes in fecal microbiota composition in PBS control animals before and after induction of MAP or SAP, and also changes in fecal microbiome composition in MAP mice treated with two different Abx regimens. As expected, both Abx treatment regimens substantially reduced the detectable bacterial 16S rDNA readouts to an equally low level (Fig. S1D). However, we observed significantly higher relative abundance of *Bifidobacterium, Akkermansia*, and *Enterococcus* spp. in the long-term Abx-treated mice (MAP resistant), but not those treated with high-concentration Abx by oral gavage (MAP susceptible). This finding suggests that Abx administration in the water over 3 weeks somehow resulted in accumulation of distinct commensal bacterial taxa even in the context of global microbiome depletion (Figure 1f,g). Consistent with this, *Bifidobacterium* was also found to be the most abundant in the PBS control mice, and significantly diminished in the MAP and SAP groups (Figure 1h,i).

### Colonization with Bifidobacterium spp. protects the host from MAP and SAP

**Figure 2.**
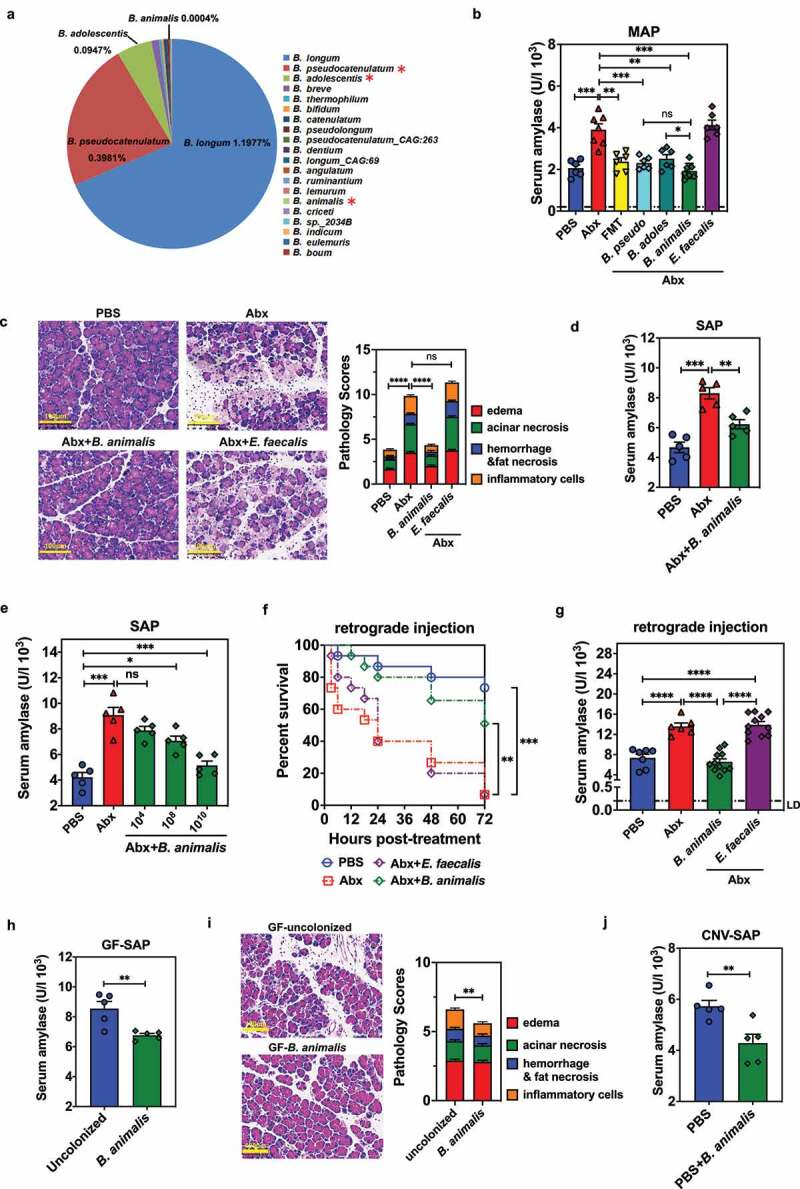
Colonization by *Bifidobacterium* spp. is protective against MAP and SAP. (a) Compositional analysis of *Bifidobacterium* genus in fecal samples collected from MAP mice administered Abx via driving water (n = 4). Figure depicts the top 20 *Bifidobacterium* spp. detected by deep sequencing. The percentage indicates the proportion of the bacteria in the total intestinal flora. (b) Serum amylase concentration of MAP mice treated with PBS, Abx, or fecal microbiota transplantation (FMT), or Abx-treated mice colonized with *B. pseudocatenulatum, B. animalis, B. adolescentis* or *Enterococcus faecalis* at 12 h post MAP modeling. (c) Pancreatic histopathology and pathology scores from mice treated with PBS, Abx or Abx-treated mice colonized with *B. animalis* or *E. faecalis* at 12 hpt of MAP (n = 5). (d) Serum amylase activity from SAP mice treated with PBS, Abx or Abx-treated mice colonized by *B. animalis* at 12 hpt (n = 5). (e) Serum amylase concentration in SAP mice treated with PBS, Abx or Abx-treated mice colonized with different doses of *B. animalis*. (f) Survival kinetics in surgical SAP model. Survival was observed for 72 h following retrograde injection of 5% sodium taurocholate into the pancreatic-bile duct in mice treated with PBS, Abx or Abx-treated mice colonized by *B. animalis* or *E. faecalis* (n = 6–14). (g) Serum amylase level of mice from (f) at 6 h post-retrograde injection. (h) Serum amylase activity in uncolonized or *B. animalis*-colonized GF mice at 12 h following SAP modeling (n = 5). (i) Pancreatic histopathological changes and pathology scores of mice from (h). (j) Serum amylase activity of conventionally raised SPF mice (CNV), either treated with PBS or colonized by *B. animalis* at 12 h post-SAP induction (n = 5). Data are from two independent experiments, and *P* values were determined by unpaired two-tailed Student’s t-test. Broken lines indicate the limit of detection (LD) of the assay. *: *P* < .05; **: *P* < .01; ***: *P* < .001; ****: *P* < .0001; ns: not significant.

Since the taxonomic analysis hinted that *Bifidobacterium* could possibly impact the onset and severity of AP, we performed high-throughput amplicon sequencing of the full-length 16S rDNA from 4 representative fecal samples from MAP mice who had received prolonged Abx in water. Species-level analysis revealed that *Bifidobacterium* genus in the intestinal microbiome of these mice was dominated by *B. longum* (~1.2% of total commensal bacteria) (Figure 2a). To further explore the role of *Bifidobacterium* in AP protection, we chose to use *B. pseudocatenulatum* (abbreviated as *B. pseudo), B. adolescentis* (abbreviated as *B. adoles*), the two commonly identified species in the enriched *Bifidobacterium* profile (Figure 2a), and *B. animalis*, a well-characterized probiotic that has been proven beneficial for a variety of metabolic and infectious diseases. *Enterococcus faecalis*, a common intestinal commensal bacterium, was included in the protection study in order to determine the role of specific *Bifidobacterium* species in AP protection.

All three *Bifidobacterium* isolates were similarly able to repopulate the gut microbiota of Abx-treated mice (Fig. S1E), whereas colonization by *B. animalis* achieved optimal efficacy in decreasing the serum amylase concentration and ameliorating pancreatic pathological changes (Figure 2b,c and Fig. S1G). On the contrary, *E. faecalis* colonization had no protective effect, even though it also colonized mice efficiently (Figure 2b,c and Fig. S1E). Due to its optimal performance in preventing MAP, we chose to focus on *B. animalis* in the subsequent studies. *B. animalis* colonization greatly diminished serum amylase in a dose-dependent manner (Figure 2d,e and Fig. S1F), and alleviated pancreatic pathological changes in the caerulein plus LPS-induced SAP model (Fig. S1H). Using the more severe surgical AP model (induction by retrograde injection of 5% sodium taurocholate into the pancreatic-bile duct), *B. animalis* (but not *E. faecalis*) colonization improved survival and lowered serum amylase (Figure 2f,g).

Furthermore, in GF mice with SAP (caerulein plus LPS-induced model), greater serum amylase and more severe pancreatic damage were detected in uncolonized GF mice than in those colonized by *B. animalis* (Figure 2h,i), suggesting these phenotypes were not due to the residual Abx-resistant bacteria in our Abx treatment model. Additionally, conventionally housed (CNV) B6 mice colonized by *B. animalis* with SAP (caerulein plus LPS-induced model) displayed not only lower serum amylase but also less pancreatic pathological damage (Figure 2j and Fig. S1I). Collectively, these data indicate that prophylactic administration of *B. animalis* is capable of ameliorating pancreatitis of different severity, and the suppression of AP by various *Bifidobacterium* spp. represents a broader mechanism by which this particular bacterial community is able to influence the host inflammatory responses.

### B. animalis-associated and exogenous lactate protects host from MAP and SAP

**Figure 3.**
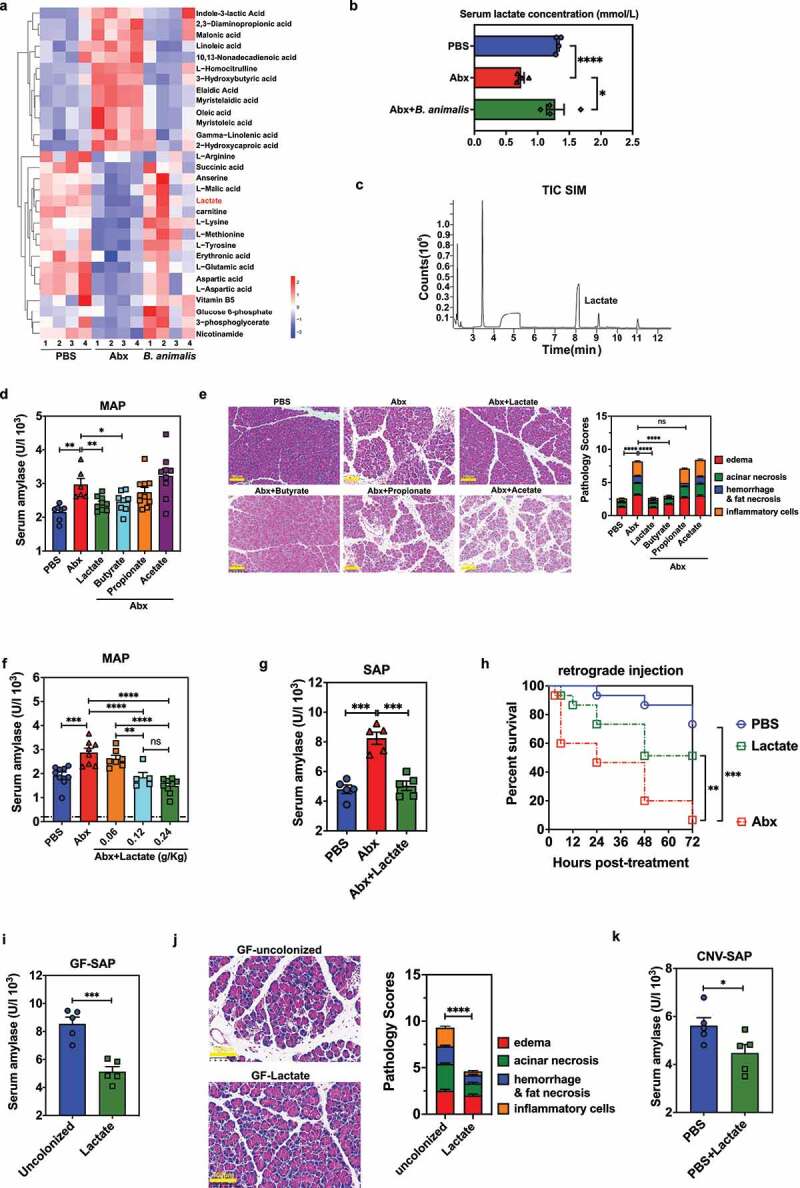
*B. animalis* colonization and exogenous lactate administration protect the host from MAP and SAP. (a) Heatmap showing differential metabolites in serum of individual animals treated with PBS, Abx or Abx-treated mice colonized by *B. animalis* at 12 h post-SAP induction. (b) Absolute lactate concentration in serum samples collected from mice from (a). (c) The supernatant of *B. animalis* cultures was analyzed by liquid chromatography coupled with tandem mass spectrometry (LC-MS/MS). (d) Serum amylase concentration, (e) pancreatic histopathological changes and pathology scores of mice treated with PBS, Abx or Abx-treated mice administered either lactate (0.24 g/kg), acetate, propionate or butyrate at 12 h post-induction of MAP (n = 6–10). (f) Serum amylase activity in mice treated with PBS, Abx or Abx-treated mice given different doses of lactate at 12 h post-induction of MAP. (g) Serum amylase level of mice treated with PBS, Abx or Abx-treated mice receiving lactate gavage (0.24 g/kg) at 12 h post-induction of SAP (n = 5). (h) Survival kinetics in surgical SAP model. Survival was observed for 72 h following retrograde injection of 5% sodium taurocholate into the pancreatic-bile duct in mice treated with PBS, Abx or Abx-treated mice receiving lactate gavage (0.24 g/kg) (n = 13–14). (i) Serum amylase level, (j) histopathological changes and pancreatic pathology scores in untreated- or lactate-treated germ-free (GF) mice at 12 h post-induction of SAP (n = 5). (k) Serum amylase activity of conventionally raised SPF mice (CNV) treated with PBS or lactate (0.24 g/kg) at 12 h post-induction of SAP (n = 5). Data are from two independent experiments, and *P* values were determined by unpaired two-tailed Student’s t-test. Broken lines indicate the limit of detection (LD). *: *P* < .05; **: *P* < .01; ***: *P* < .001; ****: *P* < .0001; ns: not significant.

Next, we wanted to test the hypothesis that *Bifidobacterium* spp. produce specific metabolites that can protect the host from pathogenesis of AP. We performed untargeted metabolomics analysis on serum collected from PBS- or Abx-treated by oral gavage SAP mice with or without *B. animalis* colonization. The pareto-scaled principal component analysis (PCA) demonstrated significant differences in metabolomics profiles of Abx-treated mice compared with that of PBS control or *B. animalis*-colonized group (Fig. S2A). KEGG analyses revealed that the differential metabolites in PBS control mice (compared to Abx-treated mice) were mainly enriched in carbohydrate and amino acid metabolism (Fig. S2B). Of particular note, the relative abundance and absolute serum concentration of the carbohydrate metabolite lactate was significantly reduced in Abx mice and restored by *B. animalis* colonization (Figure 3a,b and Fig. S2C). Since Hoque et al. demonstrated that lactate reduces pancreatic injury in AP via suppression of TLR- and inflammasome-mediated inflammation,^8^ we prioritized lactate over other upregulated metabolites as our target for further experiments. Consistently, we analyzed the *B. animalis* cultured supernatants *in vitro* bacterial culture with LC-MS/MS and identified lactate with an average concentration of 2.4 mM (Figure 3c).

To test whether lactate plays a crucial role in protecting the host from AP induction, groups of Abx-treated mice were given lactate via oral gavage for 3 consecutive days as previously described,^21^ and subjected to caerulein-induced MAP (Fig. S2D). Because butyrate has been determined to mitigate necrotizing pancreatitis,^22^ we included it as a positive control. As expected, lactate oral gavage significantly increased the serum lactate concentration in Abx-treated mice by 3 h after the final gavage (Fig. S2E). Lactate exerted a protective effect equivalent to butyrate, with a comparable decrease in serum amylase and similar amelioration of pancreatic histopathology, whereas acetate and propionate pretreatment showed no protective effect (Figure 3d,e). Additionally, exogenous lactate oral supplementation ameliorated serum amylase activity in the MAP disease model in a dose-dependent manner (Figure 3f and Fig. S2F).

Lactate also reduced serum amylase concentration and lessened pathological changes in the pancreas during drug-induced SAP (Figure 3g and Fig. S2G), and markedly increased survival rate and decreased serum amylase level in Abx-treated mice upon sodium taurocholate retrograde injection (Figure 3h and Fig. S2H), in SAP (caerulein plus LPS-induced model), GF mice also exhibited reduced amylase concentration in serum and alleviated pathological changes in pancreas (Figure 3i,j). Furthermore, lactate administration to CNV mice with SAP (caerulein plus LPS-induced model) also resulted in improved serum amylase activity and pancreatic tissue damages (Figure 3k and Fig. S2I). Collectively, these findings from several different AP mouse models indicate that lactate, both *B. animalis*-associated and exogenous, can limit pancreatitis in the context of a perturbed or an intact microbiome.

### B. animalis and its metabolite lactate protect against AP via suppression of macrophage-mediated pancreatic and systemic inflammatory responses

**Figure 4.**
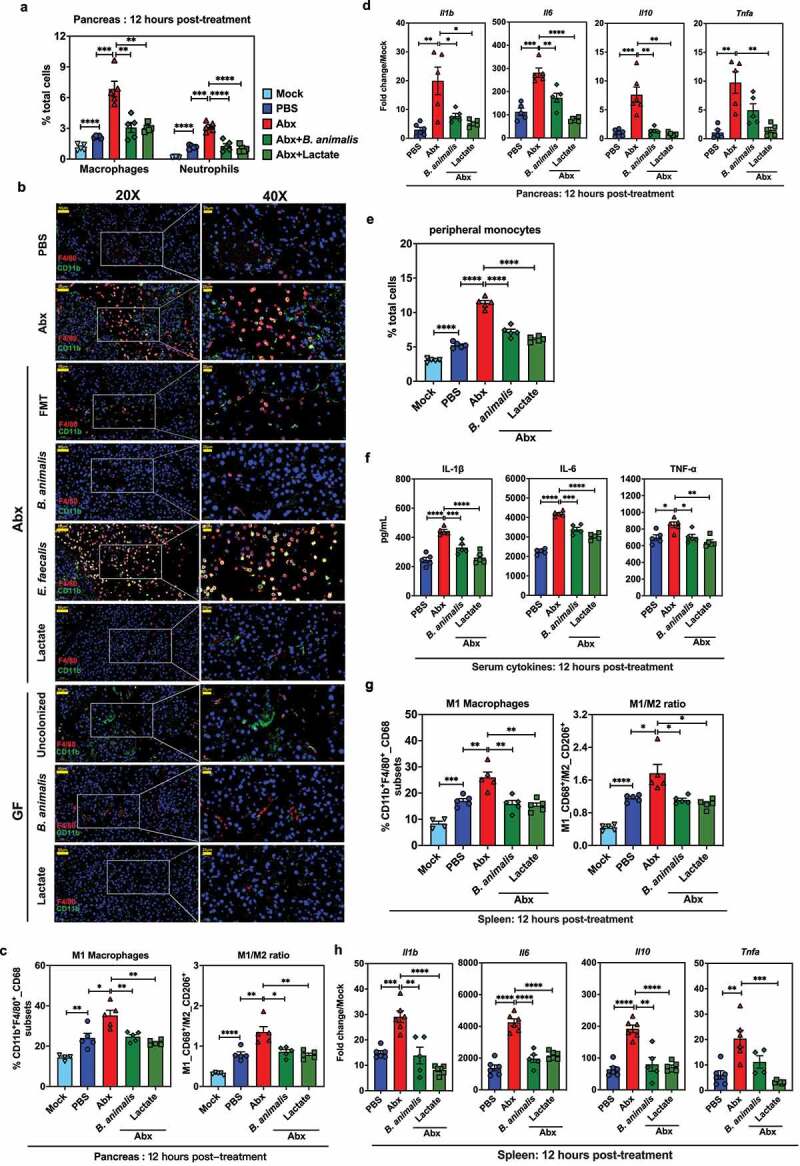
*B. animalis* and its metabolite lactate protect against AP by suppressing macrophage-mediated pancreatic and systemic inflammatory responses. (a) Frequency of pancreatic macrophages and neutrophils in mice treated with PBS, Abx, or Abx-treated mice either colonized by *B. animalis* or given lactate gavage (0.24 g/kg) at 12 h post-induction of SAP (n = 5). (b) Representative immunofluorescence images of necrotic areas within the pancreas of SAP mice treated with PBS, Abx or Abx-treated mice receiving fecal microbiota transplantation (FMT), colonization by *B. animalis* or *E. faecalis*, or lactate gavage (0.24 g/kg). In addition, germ-free (GF) mice were colonized by *B. animalis* or treated with lactate (0.24 g/kg) at 12 h post-induction of SAP (n = 5). Macrophages were double-stained with CD11b (green) and F4/80 (red) antibodies. (c, g) Frequency of CD68-expressing M1 macrophages (left panel) or M1/M2 macrophage ratio (right panel) in the (c) pancreas or (g) spleen of mock or SAP mice treated with PBS, Abx, or Abx-treated mice colonized by *B. animalis* or given lactate gavage at 12 h post-induction (n = 5). (d, h) Relative mRNA expression of *Il1b, Il6, Il10* and *Tnfa* in the (d) pancreas or (h) spleen of mock or SAP mice treated with PBS, Abx, or Abx-treated mice colonized by *B. animalis* or given lactate gavage at 12 h post-induction (n = 5–6). Fold change is relative to respective mock mice. (e) Frequency of monocytes in PBMCs and (f) serum expression (ELISA) of IL-1β, IL-6 and TNF-α in mock or SAP mice treated with PBS, Abx or Abx-treated mice colonized by *B. animalis* or given lactate gavage (0.24 g/kg) at 12 h post-induction (n = 5). Data are from two independent experiments, and *P* values were determined by unpaired two-tailed Student’s t-test. *: *P* < .05; **: *P* < .01; ***: *P* < .001; ****: *P* < .0001.

Macrophages and neutrophils drawn to the site of pancreatic injury by DAMPs are believed to be the source of systemic inflammatory responses.^23^ Thus, we first used flow cytometry to study infiltration of macrophages and neutrophils in the pancreas of mice treated with PBS, Abx, either colonized by *B. animalis* or pretreated with lactate (Fig. S3A, B). There was a profound reduction of infiltrating macrophages and neutrophils in the *B. animalis*-colonized or lactate-pretreated mice compared to those receiving Abx alone (Figure 4a). IFA revealed significantly more macrophage (CD11b^+^/F4/80^+^) and neutrophil (CD11b^+^/Ly6G^+^) signal in the pancreatic sections of Abx-treated and *E. faecalis*-colonized mice compared with the PBS, FMT, *B. animalis*-colonized or lactate-pretreated Abx mice (Figure 4b and Fig. S3C). A similar pattern of macrophage and neutrophil staining was observed in *B. animalis*-colonized or lactate-pretreated GF mice compared with untreated GF controls (Figure 4b and Fig. S3C).

Further, *B. animalis* colonization or lactate pretreatment caused a marked decreased in frequency (10.5% or 13.5%, respectively) of CD68-positive M1 macrophages in the pancreas of Abx-treated mice during SAP (Figure 4c). Since Colegio et al. demonstrated that lactate produced by aerobic or anaerobic glycolysis of tumor cells can induce expression of vascular endothelial growth factor and M2-like polarization of tumor-associated macrophages,^24^ we also measured the CD206-expressing M2 macrophages in the pancreas. *B. animalis* colonization or lactate pretreatment did not alter the frequency of M2 macrophages compared with mice receiving Abx alone, although AP induction did cause downregulation of M2 macrophages compared to mock mice (Fig. S3D). *B. animalis* colonization or lactate pretreatment significantly decreased the ratio of M1/M2 macrophages in the pancreas of Abx-treated mice (Figure 4c), indicating that they restrict AP by suppressing M1 polarization of pancreatic macrophages after disease induction. There was also significantly lower expression of pancreatic proinflammatory cytokine genes including *Il1b, Il6, Il10* and *Tnfa* in *B. animalis*-colonized or lactate-pretreated Abx mice (Figure 4d), suggesting an important role for *B. animalis*/lactate in dampening pancreatic inflammatory responses.

During SAP, a vicious cycle is driven by activated monocytes in circulation and macrophages in distant organs.^23^ *B. animalis* colonization or lactate administration decreased the frequency of monocytes in peripheral blood mononuclear cells of Abx mice by 1.6-fold or 2-fold, respectively, and reversed the differences in serum cytokine profile (Figure 4e, f).

Consistent with the results of these studies, we discovered that *B. animalis* colonization or lactate administration significantly impaired the upregulation of bulk and activated M1 macrophages, greatly lowering the M1/M2 ratio and decreasing expression of *Il1b, Il6, Il10*, and *Tnfa* in the spleen in Abx-treated mice at 12 hours following SAP induction (Figure 4g,h and Fig. S3E, F). In summary, *B. animalis* and microbial metabolite lactate both protected mice from AP by suppressing the local and systemic inflammatory responses related to macrophage stimulation.

### Prevention of macrophage-mediated inflammation during AP by B. animalis colonization or lactate administration requires TLR4-MyD88

As lactate can restrict AP with or without intestinal microbiota, we hypothesized that lactate may suppress the inflammatory response in macrophages in a cell-intrinsic manner. Thus, we extracted peritoneal macrophages from PBS or Abx-treated mice, pretreated them with lactic acid and stimulated with LPS *ex vivo*. Indeed, the expression of *Il1b, Il6*, and *Tnfa* transcripts was significantly reduced by a similar magnitude (Fig. S4A), indicating that the inflammatory response in macrophages was impaired in a cell-intrinsic manner following lactate administration.

**Figure 5.**
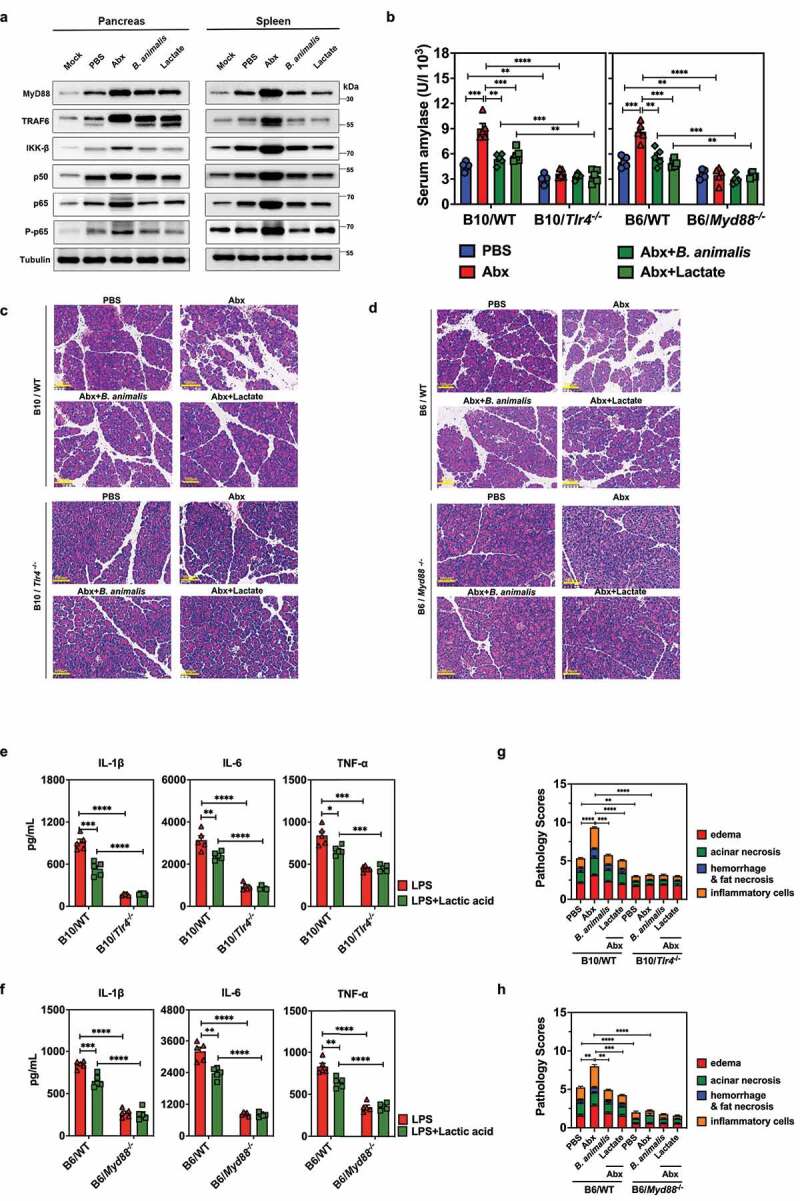
Relief of macrophage-mediated inflammation during AP by *B. animalis* colonization or lactate administration requires TLR4-MyD88 signaling. (a) Representative western blot showing expression of NF-κB signaling pathway-related molecules in the pancreas and spleen of mice treated with PBS, Abx, or Abx-treated mice colonized by *B. animalis* or given lactate gavage at 12 h post-induction of SAP (All experiments were repeated independently three times. one representative experiment was shown.) (b) Serum amylase activity, (c, d) pancreatic tissue lesions and (g, h) pathology scores of *Tlr4^−/−^* or *Myd88^−/−^* mice or their respective wild-type (WT) parent strain, treated with PBS, Abx or Abx-treated mice colonized by *B. animalis* or given lactate gavage at 12 h post-induction of SAP (n = 5). Expression (ELISA) of IL-1β, IL-6 and TNF-α in peritoneal macrophages extracted from (e) *Tlr4^−/−^* or (f) *Myd88^−/−^* mice or their respective WT parents. Cells were pretreated or not with lactic acid (15 mM), and subsequently stimulated with LPS (200 ng/mL). Data are from two independent experiments, and *P* values were determined by unpaired two-tailed Student’s t-test. *: *P* < .05; **: *P* < .01; ***: *P* < .001; ****: *P* < .0001.

A previous study reported that the NF-κB pathway was activated when bone marrow-derived macrophages were co-incubated with acini, and transcriptome analysis indicated that upstream TLR signaling pathways were activated by DAMP signals arising from necrotic acinar cells.^7^ When we evaluated the expression of inflammatory response genes associated with the TLR and NF-κB signaling pathways in our SAP mouse model, we discovered that transcripts from *Tlr4, Myd88, Traf6, Ikkb, P50* and *P65*, but not *Tlr1* or *Tlr9* were significantly decreased in the pancreas and spleen of *B. animalis*-colonized or lactate-treated Abx mice compared to mice receiving Abx alone (Fig. S5). Reduced protein expression was confirmed by western blot (Figure 5a), indicating that *B. animalis* colonization or lactate administration diminished TLR- and NF-κB-associated inflammatory responses locally and systemically during SAP.

To study the functional role of TLR signaling in the *B. animalis*- or lactate-mediated protection against AP, we established a caerulein-induced SAP model in PBS- or Abx-treated *Tlr4^−/−^* or *Myd88^−/−^* mice and their respective wild-type (WT) parents C57BL/10JGpt (referred to as B10/WT) and B6/WT mice. Consistent with our other AP models, serum amylase concentration and pancreatic histopathological changes were markedly augmented in B10/WT and B6/WT mice treated with Abx alone. This pathology was reduced to PBS control levels by *B. animalis* colonization or lactate pretreatment. However, there were no signs of pathology induced by caerulein in the B10/*Tlr4^−/−^* or B6/*Myd88^−/−^* mice (Figure 5b-d). Correspondingly, pancreatic infiltration by macrophages and neutrophils and proinflammatory cytokine expression were equivalent among B10/*Tlr4^−/−^* or B6/*Myd88^−/−^* mice, regardless of Abx treatment, *B. animalis* colonization or lactate pretreatment (Fig. S6).

Furthermore, we observed that secretion of IL-1β, IL-6, and TNF-α was greatly diminished in lactic acid-pretreated peritoneal macrophages extracted from B10/WT and B6/WT mice after LPS stimulation, a phenotype not evident in peritoneal macrophages obtained from B10/*Tlr4^−/−^* and B6/*Myd88^−/−^* mice (Figure 5e,f and Fig. S4B). Overall, these data demonstrate the suppression of macrophage-associated pancreatic and systemic inflammatory responses by *B. animalis* and lactate directly correlate with activation of TLR4 and MyD88-dependent signaling during AP.

### Suppression of NLRP3 inflammasome-mediated inflammatory responses is required for B. animalis- or lactate-dependent AP protection

Growing evidence suggests that NLRP3 inflammasome activation is crucial for systemic immune responses during SAP.^7,8,11,25^ Therefore, we next studied the transcriptional profile of the NLRP3 inflammasome signaling pathway in the pancreas and spleen of Abx-treated mice either colonized with *B. animalis* or pretreated with lactate. In general, both treatments reversed the greater splenic and pancreatic transcription of *Nlrp3, Asc*, and *Casp1* observed in the mice who received Abx alone (Fig. S7). These findings were perfectly consistent with the study conducted by Hoque *et al*, which demonstrated that lactate supplementation suppresses NLRP3 inflammasome-mediated inflammation.^11^

**Figure 6.**
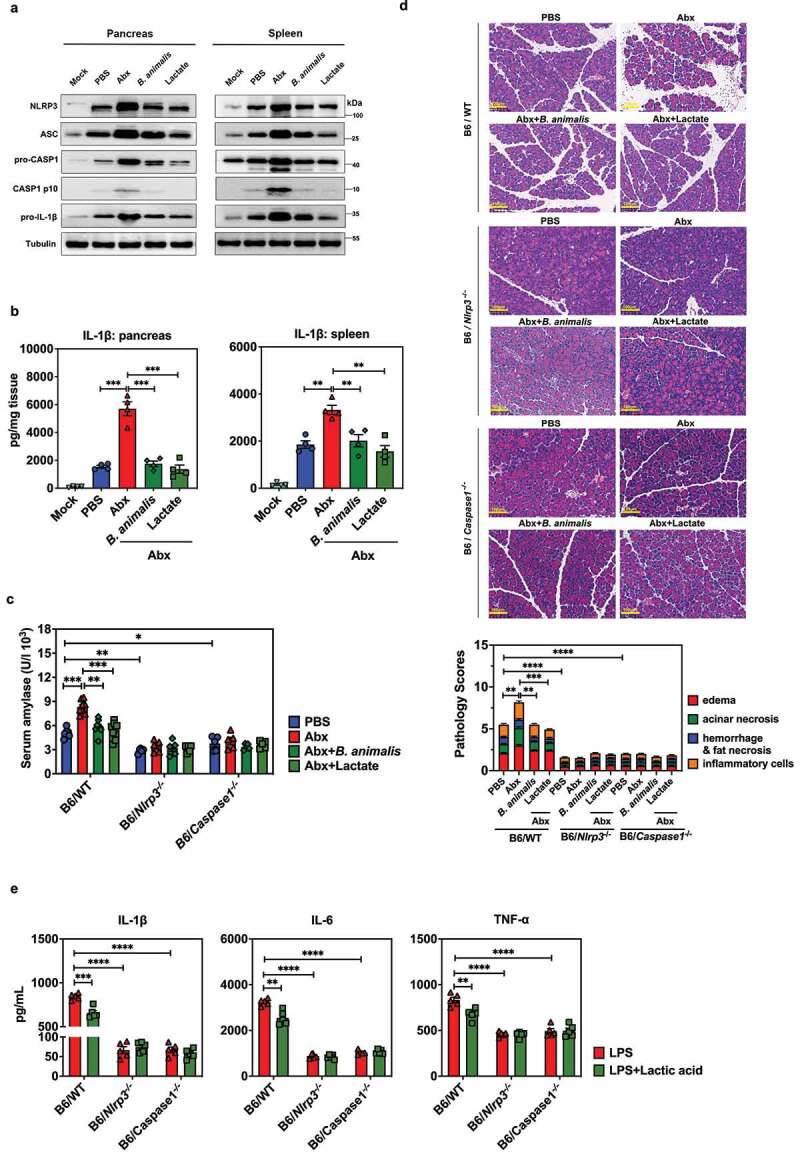
Suppression of NLRP3 inflammasome-mediated inflammatory responses is required for *B. animalis*- and lactate-dependent protection against AP. (a) Western blot showing expression of molecules related to the NLRP3 signaling pathway and (b) ELISA showing secretion of IL-1β in the pancreas and spleen of mice treated with PBS, Abx or Abx-treated mice colonized by *B. animalis* or given lactate gavage at 12 h post-induction of SAP. (c) Serum amylase concentration, (d) histopathological changes and pathology scores in the pancreas of wild-type (WT), *Nlrp3^−/−^* or *Caspase1^−/−^* mice treated with PBS, Abx or Abx-treated mice colonized by *B. animalis* or given lactate gavage (0.24 g/kg) at 12 h post-induction of SAP (n = 5). (e) Expression (ELISA) of IL-1β, IL-6 and TNF-α in peritoneal macrophages extracted from WT, *Nlrp3^−/−^* or *Caspase1^−/−^* mice. Cells were pretreated or not with lactic acid (15 mM), and subsequently stimulated with LPS (200 ng/mL). Data are from two independent experiments, and *P* values were determined by unpaired two-tailed Student’s t-test. *: *P* < .05; **: *P* < .01; ***: *P* < .001; ****: *P* < .0001.

We further corroborated the activation of NLRP3 inflammasomes by evaluating the protein level of NLRP3, ASC, pro-Caspase1, Caspase1 mature fragment (P10), pro-IL-1β and mature IL-1β in the pancreas and spleen of mock or SAP mice treated with PBS or Abx, and Abx mice colonized by *B. animalis* or treated with lactate in SAP. Indeed, depletion of the microbiota significantly augmented the NLRP3 inflammasome components and secretion of IL-1β, whereas colonization of *B. animalis* or lactate gavage greatly reduced expression of these proteins (Figure 6a,b).

To further investigate the role of the NLRP3 inflammasome in *B. animalis*- or lactate-associated protection from AP, we next repeated the experiments as previously described in *Nlrp3*^−/−^ and *Caspase1*^−/−^ mice. *B. animalis* colonization or lactate administration prevented an increase in serum amylase concentration and mitigated pancreatic tissue damage in microbiota-depleted B6/WT AP mice but not in mice lacking *Nlrp3* or *Caspase1* (Figure 6c,d). The ability of *B. animalis* colonization or lactate treatment to decrease pancreatic infiltration of inflammatory cells as well as local and systemic inflammatory responses after Abx treatment was lost in *Nlrp3*^−/−^ and *Caspase1*^−/−^ mice (Fig. S8). Similar to the experiments performed on *Tlr4^−/−^* and *Myd88^−/−^* animals, lactic acid pretreatment of peritoneal macrophages from B6/WT mice resulted in decreased expression of IL-1β, IL-6, and TNF-α after LPS stimulation, which was not seen in peritoneal macrophages from *Nlrp3*^−/−^ or *Caspase1*^−/−^ mice (Figure 6e). These data suggest that the suppression of inflammatory responses in macrophages mediated by *B. animalis* and lactate depends on NLRP3 inflammasome signaling.

### Pancreatitis patients exhibit a decreased fecal abundance of Bifidobacterium that was inversely correlated with the severity of their systemic inflammatory responses

**Figure 7.**
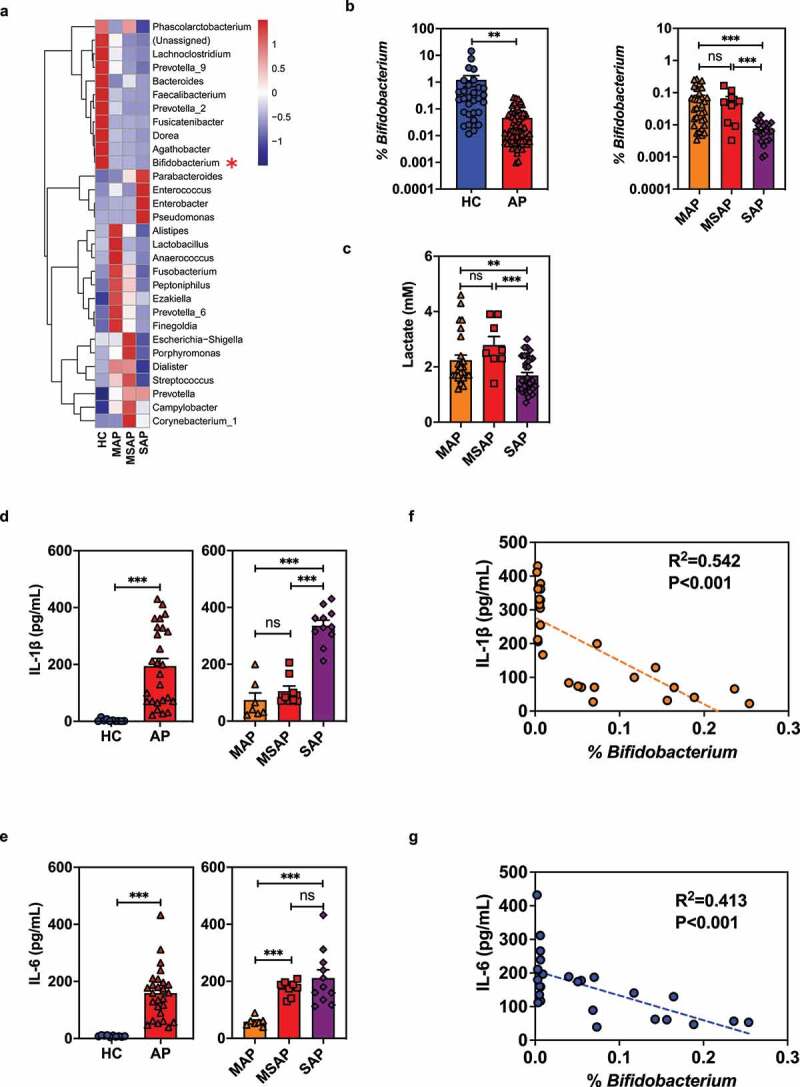
The decreased fecal abundance of *Bifidobacterium* in patients with acute pancreatitis inversely correlates with the severity of their systemic inflammatory responses. (a) The microbiota composition of human fecal samples collected from healthy volunteers (HC) and patients with clinical MAP, MSAP or SAP was analyzed at the genus level by 16S rDNA sequencing. (b) Comparison of relative abundance of *Bifidobacterium* between HC and AP patients (left panel) or among groups of patients with different levels of AP severity (right panel). (c) Serum lactate concentration in patients with AP. Differential serum expression of IL-1β (d) and IL-6 (e) between HC and AP patients (left panel) or among groups of patients with different levels of AP severity (right panel), as determined by ELISA. Correlation between fecal *Bifidobacterium* relative abundance and serum concentration of IL-1β (f) or IL-6 (g) in AP patients. Statistical significance was evaluated by one-way ANOVA. Data are from two independent experiments, and *P* values were determined by unpaired two-tailed Student’s t-test. *: *P* < .05; **: *P* < .01; ***: *P* < .001; ****: *P* < .0001; ns: not significant.

To understand the clinical relevance of our findings, we analyzed the dissimilarity of fecal microbiota composition in a total of 63 hospitalized patients (32 with MAP, 9 with moderately severe AP [MSAP] and 22 with SAP) and 30 healthy control volunteers (HC) by 16S rDNA sequencing. Remarkable differences in relative abundance of commensal bacteria were found between HC and AP patients, with a significantly lower abundance of *Bifidobacterium* observed in the AP patients (Figure 7a,b). Moreover, the abundance of the *Bifidobacterium* genus was inversely correlated with the severity of AP among the three disease groups (Figure 7b). Consistent with these results, serum lactate levels in MAP and MSAP patients were significantly higher than in SAP patients although there was no difference in serum lactate concentration between MAP and MSAP patients (Figure 7c).

Compared with HC, AP patients showed markedly higher serum expression of the inflammatory cytokines IL-1β and IL-6, and their concentrations positively correlated with disease severity in general (Figure 7d,e). There was trend (not statistically significant) for higher IL-1β in MSAP vs MAP patients, and a similar trend for higher IL-6 between SAP and MSAP patients (Figure 7d,e). Furthermore, the serum concentrations of IL-1β and IL-6 inversely correlated with the relative abundance of *Bifidobacterium* in AP patients (Figure 7f,g). Taken together, these data support a role for *Bifidobacterium* in the regulation of systemic inflammatory responses during AP.

## Discussion

Here, we describe a novel role for the *Bifidobacterium* genus in limiting immunopathogenic macrophage functions in AP and potentially other immunological disorders. Using *B. animalis* as a representative species and combining our mouse model and clinical data, we have demonstrated that: 1) *Bifidobacterium* spp. protect the host from AP by ameliorating pancreatic and systemic inflammation and immunopathological damages; 2) the *Bifidobacterium*-derived metabolite, lactate, dampens macrophage-associated inflammatory responses both locally and systemically in AP; 3) lactate limits inflammatory responses in macrophages by suppressing NF-κB and NLRP3 inflammasome activation in a TLR4-MyD88- and NLRP3-Caspase1-dependent manner during AP (Fig. S9).

Until now, most studies based on the caerulein-induced AP mouse model have reported that the microbiota promotes AP.^10,14,26^ These investigations, without exception, utilized a similar treatment regimen for depletion of intestinal microbes, one based on long-term supplementation of broad-spectrum Abx in drinking water. In the current study, we used a short-term (5-day) oral gavage with a cocktail of high-dose Abx which resulted in an equally efficient microbiota depletion (Fig. S1D). Amazingly, despite their resultant microbiota composition only differing by a few bacterial taxa (Figure 1f), mice on the two Abx regimens had remarkably disparate susceptibility to caerulein-induced AP (Figure 1d,e). The limited distinctions in microbiota composition between the two Abx regimens allowed us to target the beneficial bacterial genus *Bifidobacterium* for mechanistic experiments. Importantly, the severe disease phenotype in our caerulein induction AP model provided us the frame of reference to evaluate the protective effect exerted by bacterial colonization and related metabolites. The enrichment of *Bifidobacterium* in the long-term drinking water Abx regiment suggests that the genus is more resistant to the Abx cocktail delivered in drinking water and became one of the dominant microbial communities after the Abx-susceptible microbiota had been depleted.

Our functional studies in both Abx-treated and GF mouse models demonstrated that colonization of *Bifidobacterium* spp., particularly *B. animalis*, reduced the serum amylase concentration and ameliorated pancreatic tissue damages in caerulein-induced MAP and SAP models, and improved survival rates in a retrograde injection SAP model (Figure 2). Although subspecies of *B. animalis* have been intensively studied and recently shown to have beneficial effects for obesity, metabolic health and microbiota dysbiosis,^27,28^ our study is the first to reveal its crucial role in regulation of the pathogenesis of AP.

Metabolic profile screening revealed greatly diminished carbohydrate fermentation and amino acid metabolism in Abx-treated mice, and that *B. animalis* colonization restored serum lactate to control levels (Figure 3a and Fig. S2B, C). Exogenous lactate administration protected against AP in Abx and GF mice, as evidenced by lower serum amylase concentration, fewer histopathological changes in the pancreas and lower mortality after retrograde injection of sodium taurocholate (Figure 3). These data are in agreement with the literature that suggests lactate reduces pancreatic injury in AP via suppression of TLR- and inflammasome-mediated inflammation.^8^ Future studies with *B. animalis* mutants lacking the ability to ferment carbohydrates into lactate are needed to definitively confirm lactate’s role in *Bifidobacterium*-dependent anti-inflammatory responses.

The current study showed that *B. animalis* colonization or lactate administration significantly decreased the infiltration of neutrophils into the pancreas of Abx-treated and GF mice (Figure 4a and Fig. S3C), implicating that lactate may interfere with signals derived from injured pancreatic tissue at early phases of AP. Rapid neutrophil infiltration is usually followed by macrophage recruitment in AP.^29^ We did observe the infiltration of macrophages upon microbiota depletion, especially proinflammatory, activated M1 macrophages within the necrotic areas of the pancreas, though *B. animalis* colonization or lactate administration restored this to control levels (Figure 4a-c). Similar patterns were observed in GF mice following *B. animalis* colonization or lactate administration (Figure 4b), although AP induction caused less severe pancreatic infiltration by neutrophils and macrophages in GF mice compared with CNV mice (data not shown), supporting that GF mice are truly less sensitive to caerulein-induced AP, as previously described.^14^

Furthermore, the upregulation of peripheral monocytes, serum cytokine expression and splenic activated bulk and M1-macrophages in Abx-treated mice were reverted by *B. animalis* colonization or lactate pretreatment (Figure 4e-h), indicating that SIRS exacerbated by macrophage activation in distal organs during SAP can be efficiently mitigated by lactate. Our findings are consistent with a recent publication that demonstrated how infiltrating macrophages within areas of pancreatic necrosis secrete proinflammatory cytokines to develop SIRS, and at the same time induce a parallel compensatory anti-inflammatory response syndrome (CARS) of the adaptive immune system, which drives M2-macrophage polarization.^7^

Our study utilized a panel of knockout mice genetically deficient in key sensors and mediators, identifying a mechanism by which *Bifidobacterium* and the microbial metabolite lactate suppress caerulein-induced pancreatic and systemic inflammatory responses through a pathway that requires TLR4 and MyD88 signaling as well as an NLRP3- and Caspase1-dependent inflammasome pathway in macrophages (Figure 5e,f and Figure 6e). Our model perfectly matches the study conducted by Hoque et al., which determined a crucial role for lactate in suppressing TLR4- and NLRP3 inflammasome-mediated inflammation in AP via G_i_-protein-coupled receptor 81 (GPR81).^8^ Thus, additional studies are needed to evaluate whether or not GPR81 also mediates the protective anti-inflammatory effects of *Bifidobacterium* and lactate under AP *in vivo*.

Finally, our clinical data was consistent with the mouse models, showing an inverse relationship between the relative abundance of gut *Bifidobacterium* and AP severity (Figure 1h,i and Figure 7b), suggesting a potential role for *Bifidobacterium* as a prognostic microbial marker for AP outcome. Furthermore, increased serum IL-1β and IL-6 were found to be inversely related to abundance of intestinal *Bifidobacterium*, matching the findings reported by Tan et al., and suggesting that during the development of AP, dysbiosis of the gastrointestinal tract may contribute to an increase in severity of SIRS.^15^

One limitation of the clinical study is that the large number of clinical variables in patients with AP might affect their microbiome composition, which represents a major hurdle to the interpretation of disease causality for human microbiota alteration. Moreover, caution must be taken when substituting probiotics such as *Bifidobacterium* in AP, as Pushalkar et al., suggested that microbiota reconstitution with *B. pseudologum* accelerated oncogenesis in Abx-treated KPC mice, which express mutant intra-pancreatic Kras and p53.^30^ Further, considering the cell surface lactic acid receptor GPR81 is crucial to the survival of cancer cells, it is necessary to assess the appropriate, effective dose of lactate to be used, which is usually administrated in the form of lactated Ringer’s solution.^23^

In conclusion, our detailed functional study demonstrates how macrophages and inflammatory signaling pathways are modulated by the commensal *Bifidobacterium* to limit inflammatory responses locally and systemically, reducing subsequent immunopathology. The finding that *B. animalis* and its associated metabolite lactate can ameliorate AP may shed light on the heterogeneity of clinical outcomes and drive the development of more efficacious therapeutic interventions for pancreatitis, and potentially other inflammatory disorders.

## Materials and methods

### Antibodies and reagents

The following antibodies were used for immunoblotting, immunofluorescence, and flow cytometry analysis: anti-Caspase-1 (ab179515; Abcam), anti-NLRP3 (ab263899; Abcam), anti-ASC (sc514414; Santa Cruz), anti-NF-κB P50 (ab32360; Abcam), anti-NF-κB P65 (cst6956; Cell Signaling Technology), anti-phospho–NF-κB P65 (cst3033; Cell Signaling Technology), anti-MyD88 (ab219413; Abcam), anti-IKK-β (ab124951; Abcam), anti-TRAF6 (ab33915; Abcam), anti-GPR81 (ab106942; Abcam), anti-tubulin (11224; Proteintech), anti-pro-IL-1β (cst12242; Cell Signaling Technology), anti–F4/80-PE (123110; BioLegend), anti–CD11b-BV421 (101236; BioLegend), anti–CD68-APC (137007; BioLegend), anti–CD206-AF647 (141712; BioLegend), anti–Ly6G-FITC (11–9668-80; eBioscience), anti-CD16/32 (14–0161-82; eBioscience), anti-CD11b (2185-1-ap; Proteintech), anti-F4/80 (cst70076; Cell Signaling Technology), anti-Ly6G monoclonal antibody (65140-1-ig; Proteintech).

The following reagents were used for this study: caerulein (C9026-1 MG; Sigma, Munich, Germany), ELISA kit for IL-1β, IL-6 and TNF-α (Lianke), alpha-amylase determination kit (BIOSINO, Beijing, China), PBMC isolation kit (LTS1092; TBD), LPS from *Escherichia coli* O55:B5 (Sigma, Germany), L-lactate assay kit (ab65330; Abcam), sodium L-lactate (L7022; Sigma), L-lactic acid (L1750; Sigma).

### Animals

C57BL/6 J WT mice and homozygous *Tlr4*-deficient (referred to as *Tlr4*^−/−^) mice which were backcrossed to a C57BL/10 J background were purchased from the Model Animal Research Center of Nanjing University (Nanjing, China). Homozygous *Caspase1*-deficient (referred to as *Caspase1*^−/−^) mice were a kind gift from Dr. Rongbin Zhou (University of Science and Technology of China, Anhui, China). Homozygous *Nlrp3*-deficient (referred to as *Nlrp3*^−/−^) mice were a kind gift from Dr. Anding Zhang (Huazhong Agricultural University, Wuhan, China). Homozygous *Myd88*-deficient (referred to as *Myd88*^−/−^) mice were a kind gift from Dr. Xiaoyu Hu (Tsinghua University, Beijing, China). All studies were performed using mice at 6 to 8-weeks-of-age that weighed between 18 and 23 g. All specific-pathogen-free (SPF) mice were housed in a sterile environment with an average temperature of 22°C and a standard 12 h/12 h light/dark cycle (7:00 am-7:00 pm) in the animal experimentation center of Zhejiang University. All animal experiments were conducted in accordance with a protocol (#117113) approved by the Institutional Animal Care and Use Committee of Zhejiang University.

### Bacterial isolates

*B. pseudocatenulatum* (ATCC27919), *B. animalis* (ATCC25527) and *B. adolescentis* (ATCC15703) were purchased from BeiNa Culture Collection Co., Ltd. (Beijing, China). *E. faecalis* was kindly provided by Dr. Min Yue (Zhejiang University, Hangzhou, China) and cultured in Luria-Bertani medium (LB; Oxoid) at 37°C under aerobic conditions. Each bacterial species was quantified based on the optical density at 600 nm (OD_600_).

### Patients

A total of 63 AP patients ranging from 18 to 90 years old presenting within 7 days of onset of symptoms were enrolled from the Department of Intensive Care Unit, Sir Run Run Shaw Hospital affiliated with Medical College of Zhejiang University. All patients met the criteria for AP according to the revised Atlanta classification, and they were stratified into three groups by clinical severity: MAP, MSAP, and SAP. Exclusion criteria included those patients with chronic pancreatitis or metabolic, liver, and immunosuppressive diseases as well as cancer. Age, gender and body mass index (BMI)-matched 30 HC individuals were composed of volunteers after a routine physical examination. The HC group had the following inclusion criteria: free of chronic metabolic, cardiovascular or gastrointestinal diseases; not pregnant; no medical treatment influencing intestinal function. Both patients and healthy subjects who had received antibiotics or probiotics in the 4 weeks prior to sample collection were excluded from the study. Written informed consent was obtained from each participant following protocols approved by the institutional review boards of Sir Run Run Shaw Hospital affiliated to Medical College of Zhejiang University. Demographics and clinical variables were collected during clinic visits.

For human studies, written informed consent was obtained from all subjects before the study protocol. All human experiments were conducted in accordance with a protocol (#20200224-31) approved by the Medical Ethics Committee of Sir Run Run Shaw Hospital. Table S1 describes the baseline demographic and clinical characteristics of AP patients.

### Isolation and treatment of splenocytes, pancreatic cells, peritoneal macrophages and PBMCs in mice

Spleens were dissected, and single-cell suspensions of splenocytes were generated by grinding through a 70-μm strainer. Erythrocytes were lysed with red blood cell lysis buffer (RCLB; HyClone), and remaining cells were resuspended in PBS supplemented with 2% fetal bovine setum and 1 mM EDTA.

Samples of pancreas were dissected, minced and washed twice with 10 mL Hank’s Balanced Salt Solution containing 10% FBS. Tissue was resuspended in 10 mL HBSS containing 10% FCS and 2 mg/mL collagenase (type IV; Sigma Chemical Co. St. Louis, MO), shaken continuously in a 37°C water bath (100 cycles/min) and vortexed for 20 seconds. Cellular suspensions were centrifuged and resuspended in 0.5 mL pancreas ACK Lysis Buffer to remove red blood cells. Resultant cells were immediately centrifuged, washed 3 times with HBSS, and passed through a 70-µm filter to produce a single-cell suspension of pancreatic cells.

Peritoneal macrophages were isolated as previously described.^11^ PBMCs were isolated by density gradient centrifugation using a PBMC isolation kit (TBD, Tianjin, China).

### Abx treatment, FMT, and bacterial colonization

Mice were given an Abx cocktail composed of ampicillin 33.2 mg, neomycin 33.2 mg, metronidazole 33.2 mg, and vancomycin 16.7 mg daily for 5 days via oral gavage. After the 5th day of oral gavage, Abx were added to the drinking water at a concentration of 1 g/L each for ampicillin, neomycin, and metronidazole and 500 mg/L for vancomycin. Fecal samples collected from microbiota-depleted mice at 5 days post-treatment were homogenized, plated on BHI agar with 10% sheep blood, and cultured under anaerobic conditions at 37°C for 2 days followed by incubation under aerobic conditions at 37°C for 1 day to confirm efficient microbial depletion. Animals were maintained on Abx- or PBS-containing water for the duration of the experiment.

For FMT experiments, feces were collected under 20% O_2_ condition and were used immediately after fresh harvest. 200 mg of pooled fecal pellets were homogenized with sterile silica beads in 1.5 mL PBS at 45 Hz for 1 min and filtered with 70-μm strainers. Mice were orally administered Abx as described above, except Abx were discontinued on day 6, and mice were individually subjected to gavage with 150 μL filtered stool homogenates.

For bacterial colonization experiments, mice were subjected to gavage with 10^10^ CFU of *E. faecalis, B. pseudocatenulatum, B. animalis* or *B. adolescentis* in 150 μL PBS after 5 consecutive days of oral Abx administration.

Two days after FMT or bacterial colonization (3 days after final oral Abx gavage), stool samples were collected to determine the efficiency of colonization, and colonized mice were processed for pancreatitis modeling.

### qRT-PCR

RNA was isolated from tissues or cells using TRIzol reagent (Invitrogen) per the manufacturer’s instructions. Gene expression levels were determined via qRT-PCR (primers used for the assay are listed in Table S2) and normalized to GAPDH expression. Results are presented as fold change of gene expression in AP mice relative to that in mock animals (2^−ΔΔ*CT*^).

### Histopathology processing, scoring, and immunofluorescence assays

The pancreas was treated with 4% paraformaldehyde (PFA) and embedded in paraffin. 5-µm sections were cut and stained with hematoxylin and eosin (H&E) and semiquantitatively scored using Schmidt’s criteria by two board-certified veterinary pathologists in a double-blinded manner.^32^ The final pathology score expressed is the average of these two values.

For immunofluorescence assays (IFA), pancreatic tissue was collected from mice at 12 hours post-caerulein injection, fixed in 4% paraformaldehyde, and embedded in paraffin. Dewaxed pancreatic sections were incubated with 3% BSA for 30 min to block nonspecific binding, stained with anti-CD11b (2185-1-ap; Proteintech) and anti-F4/80 (cst70076; Cell Signaling Technology) monoclonal antibodies, or with CD11b (2185-1-ap; Proteintech) and anti-Ly6G monoclonal antibody (65140-1-ig; Proteintech). After incubation with the primary antibody overnight at 4°C, the secondary antibody (Proteintech) was added and incubated at room temperature for 30 min. Stained sections were imaged with a Nikon Eclipse C1 vertical fluorescence microscope.

### Flow cytometry analysis

Splenocytes, pancreatic cells, and PBMCs were harvested for the analysis of different surface antigens in cell subsets following blockade of Fcγ receptors with anti-CD16/32 (eBioscience). Fluorescent antibodies used included those specific to CD11b, CD68, CD206, Ly6G and F4/80 (BioLegend).

### Western blots

Tissues were homogenized in ice-cold buffer containing 20 mM HEPES (pH 7.0), 150 mM NaCl, 1 mM EDTA, 1% Triton X-100, Proteinase Inhibitor Cocktail (Roche Applied Sciences), and Halt Phosphatase Inhibitor Cocktail (Thermo Scientific) for 30 s. The samples were sonicated through the Cell Ultrasound Cruizer (Thermo Scientific) for 10 s and centrifuged at 15,000 rpm for 10 min, and total proteins were recovered in the supernatants. The supernatants were collected for BCA protein kit (Beyotime Institute of Biotechnology, Nanjing, China). Samples (40 μg protein per lane) were loaded on 10% SDS-PAGE gels. After electrophoresis, the proteins were transferred to PVDF membranes (Millipore, Boston, MA, USA). Then, the membranes were blocked with 5% BSA and incubated at 4°C overnight with the appropriate primary antibodies. The membranes were washed four times for 10 min each in TBS-Tween-20, followed by incubation with secondary Ab (Fude Institute of Biotechnology, Hangzhou, China) for 60 min at room temperature. Then the membranes were detected by exposure to high ECL assay. Size estimates for proteins were obtained using m.w. standards from Bio-Rad. All experiments were performed in triplicate and carried out three times independently.

### Sodium acetate, propionate, and butyrate supplementation

Briefly, 200 mM each of sodium acetate, sodium propionate, and sodium butyrate (Sigma-Aldrich) were dissolved in drinking water of mice and replaced every 3 days. Mice received drinking water supplemented with acetate, propionate, and butyrate for 4 weeks prior to AP modeling.

### Lactate treatment

For *in vivo* experiment, mice were perfused with 0.24 g/kg body weight sodium lactate by oral gavage three days before AP modeling. For *in vitro* studies, peritoneal macrophages were pretreated with 15 mM lactic acid for 15 minutes before LPS (200 ng/mL) stimulation and tested by qRT-PCR.

### Serum amylase, lipase, and lactate measurement

Serum samples collected from animal experiments were assessed for lactate concentration by a commercial kit (ab65330; Abcam). Serum amylase concentration was determined by the Alpha-amylase Determination Kit (BIOSINO, Beijing, China). Serum lipase concentration was determined by the Lipase Activity Assay Kit (BC2340; Solarbio).

### Induction of different AP mouse models

For the MAP model, groups of mice were given 7 hourly intraperitoneal (i.p.) injections with 25 μg/kg of caerulein (Sigma). The caerulein plus LPS-induced SAP model was established by 10 hourly i.p. injections of caerulein (25 μg/kg) plus a single i.p. injection of LPS (7.5 mg/kg). A surgical SAP model was established by retrograde injection of 125 μg/kg sodium taurocholate (5%) into the pancreatic-bile duct, and survival kinetics were observed for 72 hours as described previously.^23^

### Germ-free experiments

Male C57BL/6 GF mice at 6 to 7-weeks-of-age were provided by the Experimental Animal Center of Huazhong Agricultural University, Wuhan, China. GF mice were fed in a flexible film plastic isolator. All conditions were kept sterile, and the feces and skin of GF mice were tested to verify compliance with the Chinese laboratory animal microbiological standards and monitoring (GB 14922.22–2011).

### DNA extraction, 16S rDNA sequencing, deep sequencing, and data analyses

Genomic DNA in fecal samples was extracted using the ALFA-SEQ Advanced Stool DNA Kit (Magen). The quality and quantity of DNA were measured using a NanoDrop One instrument (Thermo Fisher Scientific). Subsequently, bar-coded PCR primers targeting the V3-V4 region of bacterial 16S rDNA (forward primer, ACTCCTACGGGAGGCAGCA; reverse primer, GGACTACHVGGGTWTCTAAT) were used to generate amplicons, and multiplex sequencing of amplicons with sample-specific barcodes was performed using an Illumina Novaseq 6000 platform (paired-end 2 × 250-nucleotide reads; Guangdong Magigene Biotechnology Co., Ltd., Guangzhou, China).

Raw sequence data were filtered using fastp (version 0.14.1, https://github.com/OpenGene/fastp) with the parameters -W4 -M20 and further processed with cutadapt (https://github.com/marcelm/cutadapt/) to remove the primer sequences and generate the paired-end clean data. Subsequently, the usearch-fastq_mergepairs tool (version 10; http://www.drive5.com/usearch/) was utilized to merge the raw tags, which were later trimmed by fastp to get clean tags. The operational taxonomic units (OTUs) were then clustered using UPARSE software, with a cutoff value of 97% similarity. Each representative sequence of these OTUs was assigned using the SILVA database to annotate taxonomic information. The richness of certain commensal bacteria taxa was calculated using the usearch-alpha_div (version 10; http://www.drive5.com/usearch/) according to the OTU abundance. Based on the relative abundance of species at each classification level in otu_table, R software was used to draw the histogram, heat map, and ternary-phase diagram.

### Metabolomics profiling

LC-MS/MS analysis was conducted using an ultra-high performance liquid chromatography (1290 Infinity LC, Agilent Technologies) and quadrupole time-of-flight (AB SCIEX QQQ 5500). 5500 QTRAP (AB SCIEX) was performed in positive and negative switch mode. Peak chromatographic area and retention time were analyzed with Multiquant software.

## Statistical analysis

Statistical analyses were performed with Prism GraphPad software v 8.0. Error bars represent standard errors of the means in all figures and *P* values (the cutoff for statistical significance was P ≤ .05) were determined by unpaired, two-tailed Student’s t-test. A log-rank test was used for survival curves. One-way ANOVA was used for correlation analysis.

## Study approval

All animal experiments were conducted in accordance with a protocol (#117113) approved by the Institutional Animal Care and Use Committee of Zhejiang University. For human studies, written informed consents were obtained from all subjects before the study protocol. All human experiments were conducted in accordance with a protocol (#20200224-31) approved by the Medical Ethics Committee of Sir Run Run Shaw Hospital, affiliated with Medical College of Zhejiang University.

## Supplementary Material

Supplemental Material

## Data Availability

The data that support the findings of this study are available in figshare at https://doi.org/10.6084/m9.figshare.19534105.v1, reference number 19534105.
