# Supplementary material for: Bifidobacterium spp. and their metabolite lactate protect against acute pancreatitis via inhibition of pancreatic and systemic inflammatory responses
Source: Gut Microbes. 2022 Oct 4;14(1):2127456. doi: 10.1080/19490976.2022.2127456 (PMC9542615; doi:10.1080/19490976.2022.2127456)
Supplement: Supplemental Material [file KGMI_A_2127456_SM6553.zip › Supplementary data combined (1).docx]

**Supplementary Material**

Table S1. Baseline demographic and clinical characteristics of the patients with AP

| Characteristics | MAP | MSAP | SAP |
| --- | --- | --- | --- |
| n | 32 | 9 | 22 |
| Sex (M/F) | 24/8 | 7/2 | 12/10 |
| Age (y, mean ± SEM) | 46.53±2.09 | 48.50±5.08 | 49.82±2.76 |
| Laboratory parameters  (mean ± SEM) |  | | |
| Leukocyte count (3.5-9.5×10^9^/l) | 11.16±0.88 | 13.72±1.22 | 14.16±1.17 |
| Lymphocyte (20.0-50.0 %) | 12.72±1.07 | 6.96±0.80 | 9.19±0.81 |
| Neutrophils (40.0-75.0 %) | 80.61±1.40 | 87.27±0.91 | 84.69±0.98 |
| Monocytes (3.0-10.0 %) | 5.78±0.43 | 5.29±0.21 | 5.31±0.34 |
| CRP (<6 mg/L) | 95.66±15.02 | 183.53±48.07 | 187.82±15.54 |
| Total bilirubin (<20.5 μmol/L) | 25.37±3.23 | 35.18±7.86 | 42.03±12.71 |
| Triglyceride(<1.70 mmol/L) | 17.92±4.19 | 17.76±9.80 | 7.20±1.91 |
| Amylase (35-135 IU/L) | 733.87±161.12 | 1117.4±365.32 | 1306.56±305.63 |
| Lipase (5.60-51.30 U/L) | 1121.83±240.78 | 1270.59±424.67 | 1526.77±513.64 |
| PCT (<0.05 ng/mL) | 2.76±1.19 | 8.47±3.49 | 3.73±1.09 |
| Improved Marshall score | 0.64±0.20 | 2.50±0.92 | 2.57±0.20 |
| APACHE-II in 24 hours | 8.75±1.05 | 14.17±1.60 | 12.55±0.95 |
| SOFA | 1.60±0.40 | 2.67±0.84 | 4.64±0.47 |
| Etiology |  | | |
| BAP | 13 | 3 | 14 |
| HLAP | 16 | 5 | 7 |
| AAP | 1 | 1 | 1 |
| IAP | 2 | / | / |
| Length of hospitalization  (d, mean ± SEM) | 10.90±1.89 | 27.50±10.50 | 37.31±6.11 |

F, female; M, male; PCT, Procalcitonin; APACHE-II in 24 hours, Acute Physiology and Chronic Health Evaluation in 24 hours; SOFA, Sequential Organ Failure Assessment; BAP, biliary AP; HLAP, hyperlipidemic AP; AAP, alcohol-induced AP;IAP, idiopathic AP.

Table S2. Primer sets used for quantitative reverse transcription-PCR

| Gene | Premier sequence |  |
| --- | --- | --- |
|  | Forward primer | Reverse primer |
| *M-Il1b* | 5'- AAGCCTCGTGCTGTCGGACC -3’ | 5'- TGAGGCCCAAGGCCACAGGT -3’ |
| *M-Il6* | 5'- CACAGAGGATACCACTCCCAACA -3’ | 5'- TCCACGATTTCCCAGAGAACA -3’ |
| *M-Il10* | 5'- CCAGAGCCACATGCTCCTAGA -3’ | 5'- GGTCCTTTGTTTGAAAGAAAGTCTTC -3’ |
| *M-Tnfa* | 5'- CATCTTCTCAAAATTCGAGTGACAA-3’ | 5'- CCAGCTGCTCCTCCACTTG -3’ |
| *M-Caspase1* | 5'- ATGGCTGACAAGATCCTGAGG-3’ | 5'- TGGGGCCCTTTTTTAGAGACAT-3’ |
| *M-Asc* | 5'- CTTGTCAGGGGATGAACTCAAAA-3’ | 5'- GCCATACGACTCCAGATAGTAGC-3’ |
| *M-Nlrp3* | 5'- TCACAACTCGCCCAAGGAGGAA-3’ | 5'- AAGAGACCACGGCAGAAGCTAG-3’ |
| *M-Gapdh* | 5'- GCCTTCCGTGTTCCTACCC-3’ | 5'- CCCTCAGATGCCTGCTTCAC-3’ |
| *M-Myd88* | 5'- ACTCCTTCATGTTCTCCATACCCTTGGT-3’ | 5'- TGCGAGTGGGGTCAGGGCG-3’ |
| *M-Traf6* | 5'- AGCCAGTCGTCCAGTGACTGC-3’ | 5'- AGAGGTGGGTCAAACTCCACATC-3’ |
| *M-Ikkb* | 5'- AACCCAGACATGTGGAGCCTGG-3’ | 5'- ATCTGGATTTCGAGGCACCAGCG-3’ |
| *M-P65* | 5'- AACGGGGCATGCGATTCCGCTA-3’ | 5'- ACTGTTCCTGGTCCTGTGTAGCCA-3’ |
| *M-P50* | 5'-ATCCCTACGGAACTGGGCAAATGTTTCA-3’ | 5'-ATCGAAATCCCCTCTGTTTTGGTTGCTCTA-3’ |
| *M-Tlr4* | 5'- AGGACTCTGATCATGGCACTGTTC-3’ | 5'- ATGTTCTTGGTTGAAGAAGGAATGTCA-3’ |
|  |  |  |
|  |  |  |

**Figure S1.** *B. aniamlis* colonization antagonizes AP-induced pancreatic tissue necrosis. (A) Serum lipase levels of SAP or MAP mice treated with PBS, Abx or FMT at 12 hours post-treatment (hpt) (n=5). (B) Serum amylase levels of non-AP mice treated with PBS and Abx (n=4). (C) 16S rDNA copies per fecal pellet as detected by qRT-PCR of the V4 hypervariable region of the 16S rRNA gene. (D) Sobs α-diversity index of 16S rRNA sequencing analysis of mouse fecal samples (n = 3 to 6). (E) Relative bacterial DNA copies per fecal pellet by qRT-PCR for Abx mice colonized with *B. pseudocatenulatum*, *B. animalis*, *B. adolescentis* or *E. faecalis*. (F) Relative *B. animalis* DNA copies per fecal pellet as detected by qRT-PCR for Abx mice colonized with different doses of *B. animalis*. (G) Pancreatic pathological sections and scores in mice treated with Abx-treated mice and Abx*-*treated mice colonized with *B. pseudocatenulatum*, *B. animalis* or *B. adolescentis* at 12 hpt of MAP (representative images, n=5). (H) Pancreatic pathological changes and scores in PBS, Abx or *B. animalis*-colonized Abx mice at 12 h post SAP induction. (I) Pancreatic pathological sections and scores in PBS-treated or *B. animalis*-colonized SAP mice at 12 hpt. Data are from two independent experiments, and *P* values were determined by unpaired two-tailed Student's t-test. Broken lines indicate the limit of detection (LD) of the assay. *: *P* < .05; **: *P* < .01; ***: *P* < .001; ****: *P* < .0001; ns: not significant.

**Figure S2.** Lactate administration ameliorates AP-induced pancreatic tissue damages. (A) The Pareto-scaled principal component analysis (PCA) demonstrated significant differences on serum metabolomics profiles of Abx-treated mice as compared with that of PBS control or *B. animalis*-colonized group (n=4). (B) KEGG analysis of different regulated metabolites between PBS and Abx mice (n=4). (C) Volcano Plot of different regulated metabolites between *B. animalis*-colonized Abx and Abx mice (n=4). (D) Schematic illustration of the MAP and SAP model by caerulein injection that was established under PBS, antibiotic microbiota depletion and lactate pretreatment. (E) Serum lactate concentrations were assessed by a fluorescent commercial L-lactate assay kit. (F) Pancreatic histopathological analysis of PBS-treated mice, Abx-treated mice and Abx-treated mice administrated with different doses (0.06, 0.12 and 0.24 g/kg) of lactate at 12 h post MAP modeling (representative images, n=5). (G) Pancreatic histopathological analysis of PBS-treated mice, Abx-treated mice and Abx-treated mice administrated with lactate at 12 h post SAP modeling. (H) Amylase level in serum of PBS (n=7), Abx (n=5) and lactate-gavaged Abx mice (n=11) at 6 h post SAP induced by retrograde injection. (I) Representative images of pancreatic damages and pathological scores in PBS- or lactate-treated CNV mice at 12 hpt of SAP (n=5). Data are from two independent experiments, and *P* values were determined by unpaired two-tailed Student's t-test. Broken lines indicate the limit of detection (LD) of the assay. *: *P* < .05; **: *P* < .01; ***: *P* < .001; ****: *P* < .0001.

**Figure S3.** *B. animalis* colonization and lactate administration restrain neutrophil infiltration into necrotic areas within pancreas during AP. Flow cytometry gating strategy for macrophages (A) and neutrophils (B) evaluation (All experiments were independently repeated three times). (C) Representative IFA images of necrotic areas within pancreas of PBS-treated mice, Abx-treated mice or Abx mice reconstituted with FMT, *B. animalis* or *E. faecalis* or Abx mice treated with lactate and uncolonized-, *B. animalis*-colonized or lactate-treated GF mice at 12 hpt of SAP (n=5). Neutrophils were double stained CD11b (red) and Ly6G (green) antibodies. (D) Expression of activation marker CD206 on CD11b^+^F4/80^+^ macrophages in pancreas of mock, PBS, Abx, *B. animalis*-colonized or lactate-treated Abx mice at 12 h post SAP induction. (E) Frequency of splenic bulk of mock or SAP mice treated with PBS, Abx, Abx plus *B. animalis* or lactate at 12 hpt (n=5). (F) Expression of activation marker CD206 on CD11b^+^F4/80^+^ macrophages in spleen of mock, PBS, Abx, *B. animalis*-colonized or lactate-treated Abx mice at 12 h post SAP induction. Data are from two independent experiments, and *P* values were determined by unpaired two-tailed Student's t-test. Broken lines indicate the limit of detection (LD) of the assay. *: *P* < .05; **: *P* < .01; ***: *P* < .001; ****: *P* < .0001; ns: not significant.

**Figure S4.** Lactate suppresses macrophage-associated inflammatory responses in a cell-intrinsic manner during AP. (A) Expression of *Il1b*, *Il6* and *Tnfa* in peritoneal macrophages isolated from mice treated with LPS and lactic acid *in vitro* by qRT-PCR. (B) Expression of *Il1b* in lactic acid-pretreated peritoneal macrophages (extracted from CNV mice) at indicated time-points following LPS stimulation *in vitro* by qRT-PCR. Data are from two independent experiments, and *P* values were determined by unpaired two-tailed Student's t-test. *: *P* < .05; **: *P* < .01; ***: *P* < .001; ****: *P* < .0001; ns: not significant.

**Figure S5.** Transcriptional profile of important molecules in TLR and NF-κB signaling pathway. Expression of *Tlr4, Myd88, Ikkb, Traf6, P50, P65* in the pancreas (A) and spleen (B) of mice treated with PBS, Abx and Abx plus *B. animalis* or lactate gavage (fold change compared to respective mock mice) at 12 hpt of SAP (n = 5 or 6). (C) Expression of *Tlr1 and Tlr9* in the pancreas (left panel) and (right panel) of PBS, Abx, *B. animalis*-colonized or lactate-administrated Abx mice at 12 h post SAP induction (n = 5 or 6). Data are from two independent experiments, and *P* values were determined by unpaired two-tailed Student's t-test. *: *P* < .05; **: *P* < .01; ***: *P* < .001; ****: *P* < .0001.

**Figure S6.** TLR4-MyD88 axis is required for *B. animalis*- and lactate-dependent protection against macrophage-associated inflammatory responses during AP. Representative IFA images of necrotic areas within pancreas of PBS-treated *Tlr4^-/-^* or *Myd88^-/-^* mice, Abx-treated *Tlr4^-/-^* or *Myd88^-/-^* mice or Abx *Tlr4^-/-^* or *Myd88^-/-^* mice reconstituted with *B. animalis* or treated with lactate at 12 hpt of SAP (n=5). (A) Macrophages were double-stained with CD11b (green) and F4/80 (red) antibodies. (B) Neutrophils were double-stained with CD11b (red) and Ly6G (green). Expression of *Il1b, Il6* and *Tnfa* in the pancreas and spleen of *Tlr4^-/-^* (C) or *Myd88^-/-^* (D) mice treated with PBS, Abx, Abx plus *B. animalis* or lactate (fold change compared to respective mock mice) at 12 hpt of SAP (n = 5 or 6). Data are from two independent experiments, and *P* values were determined by unpaired two-tailed Student's t-test. *: *P* < .05; **: *P* < .01; ***: *P* < .001; ****: *P* < .0001.

**Figure S7.** Transcriptional profile of important molecules in NLRP3 signaling pathway. Expression of *Nlrp3, Asc and Casp1* in the pancreas (A) and spleen (B) of mice treated with PBS, Abx, Abx and Abx plus *B. animalis* or lactate gavage (fold change compared to respective mock mice) at 12 hpt of SAP (n = 5 or 6). Data are from two independent experiments, and *P* values were determined by unpaired two-tailed Student's t-test. *: *P* < .05; **: *P* < .01; ***: *P* < .001; ****: *P* < .0001.

**Figure S8.** NLRP3 singaling pathway is essential for *B. animalis*- and lactate-driven amelioration on macrophage-associated inflammatory responses during AP. Representative IFA images of necrotic areas within pancreas of *Nlrp3^-/-^* and *Caspase1^-/-^* mice treated with PBS, Abx, Abx plus *B. animalis* or lactate administration at 12 h post SAP induction. (A) Macrophages were double-stained with CD11b (green) and F4/80 (red) antibodies. (B) Neutrophils were double-stained with CD11b (red) and Ly6G (green). Expression of *Il1b, Il6* and *Tnfa* in the pancreas and spleen of *Nlrp3^-/-^* (C) and *Caspase1^-/-^* (D) mice treated with PBS, Abx, Abx plus *B. animalis* or lactate (fold change compared to respective mock mice) at 12 hpt of SAP (n = 5 or 6). Data are from two independent experiments, and *P* values were determined by unpaired two-tailed Student's t-test. *: *P* < .05; **: *P* < .01; ***: *P* < .001; ****: *P* < .0001.

**Figure S9.** A model by which the *Bifidobacterium* and microbial lactate restricts pancreatic damages and subsequent SIRS by suppressing local or systemic macrophage-associated inflammatory responses through a TLR4/MyD88- and NLRP3 inflammasome-dependent pathway.
