# Supplementary figures and images for: Bifidobacterium spp. and their metabolite lactate protect against acute pancreatitis via inhibition of pancreatic and systemic inflammatory responses
Source: Gut Microbes. 2022 Oct 4;14(1):2127456. doi: 10.1080/19490976.2022.2127456 (PMC9542615; doi:10.1080/19490976.2022.2127456)

Fig. S1

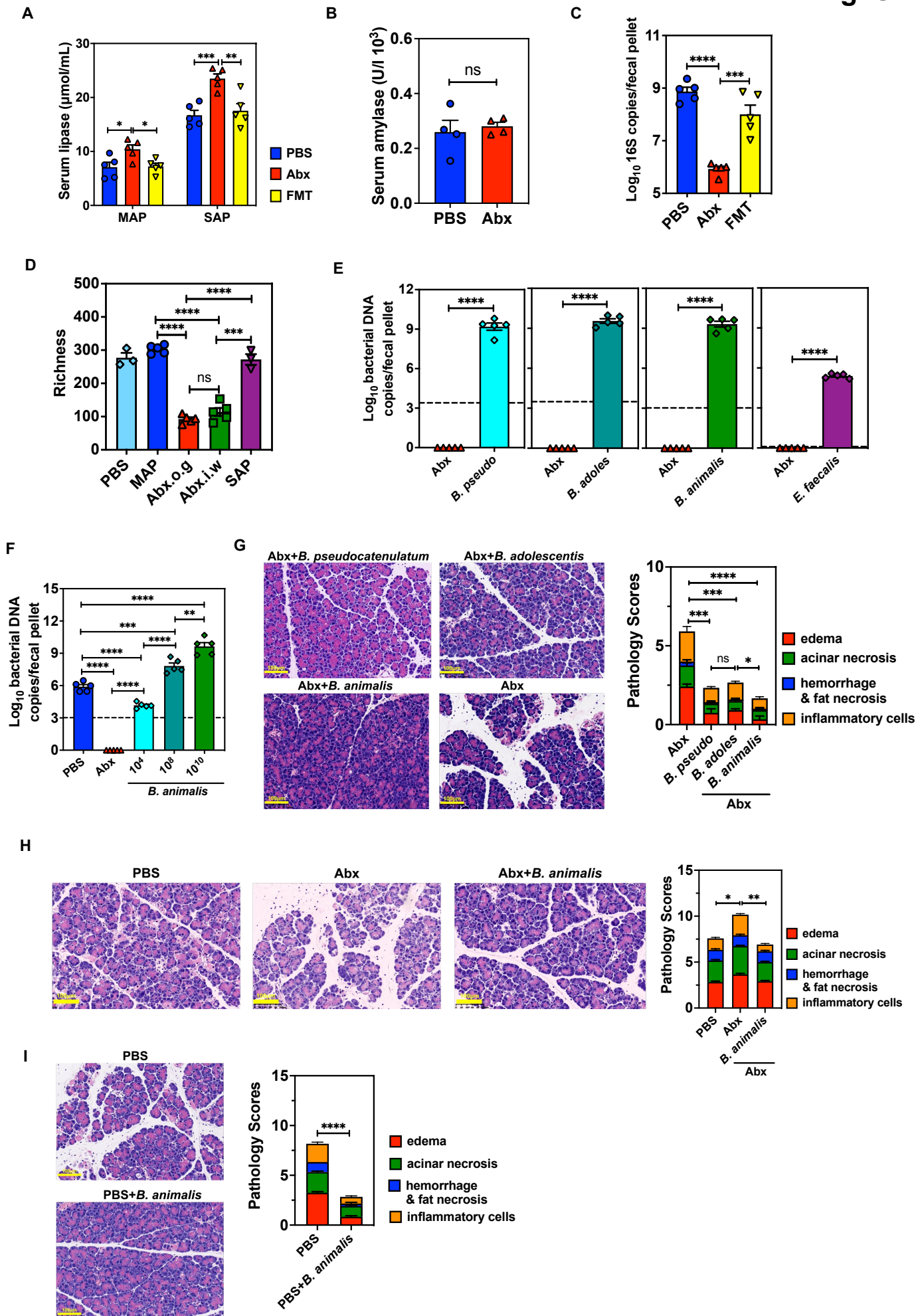

Fig. S2

A

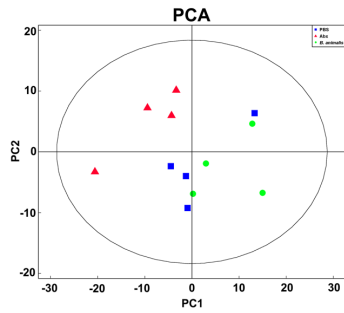

B

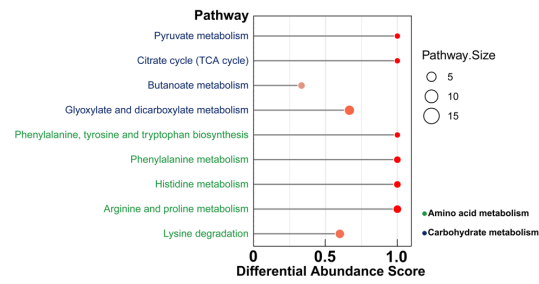

C

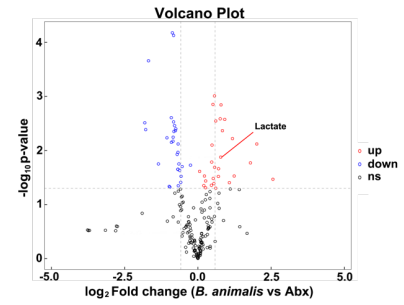

D

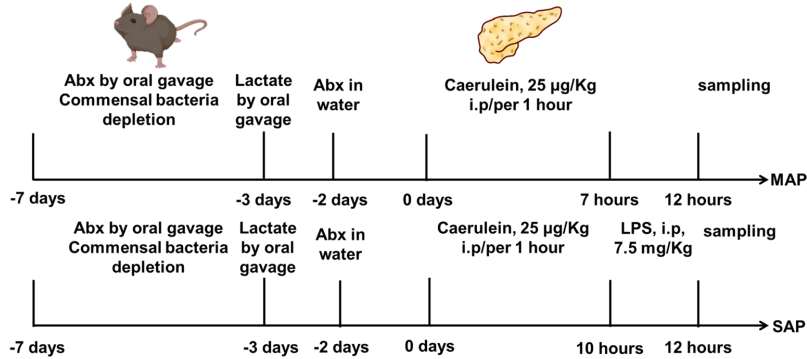

E

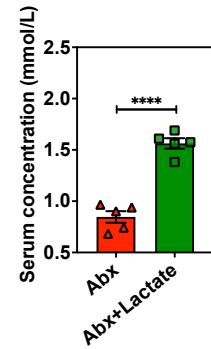

F

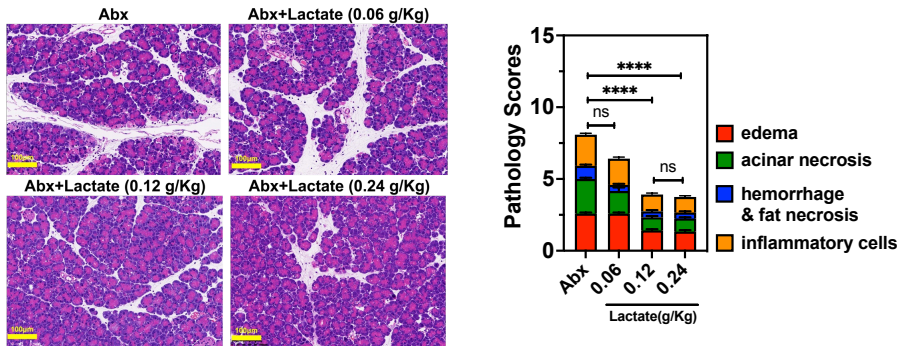

G

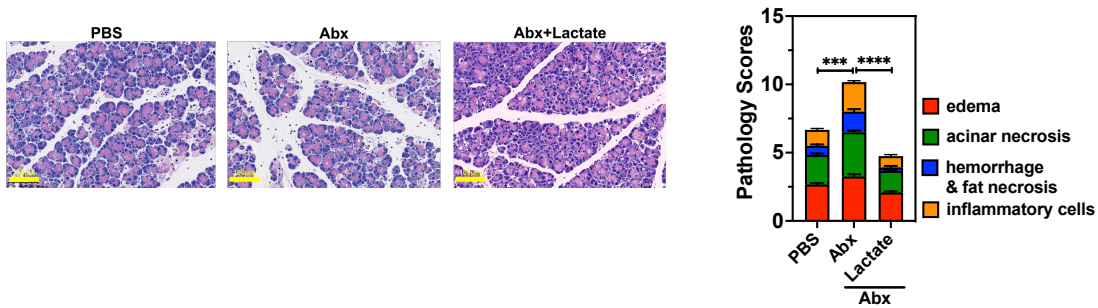

H

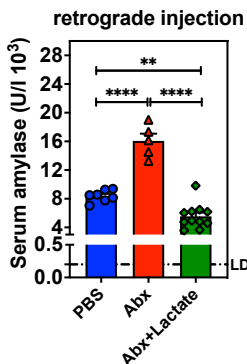

I

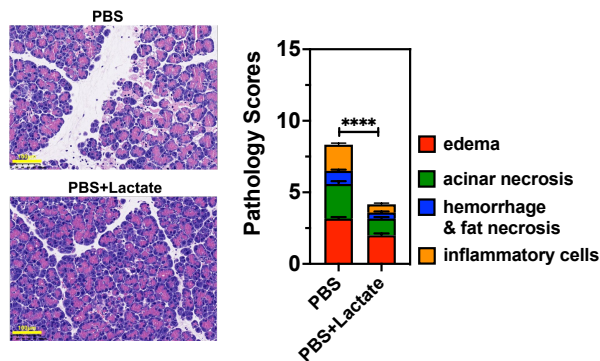

**Fig. S3**

**A**

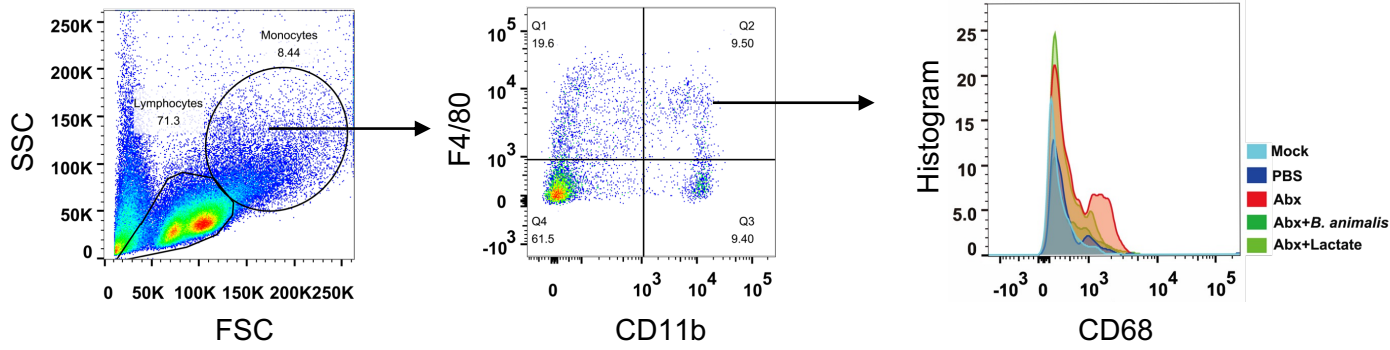

**B**

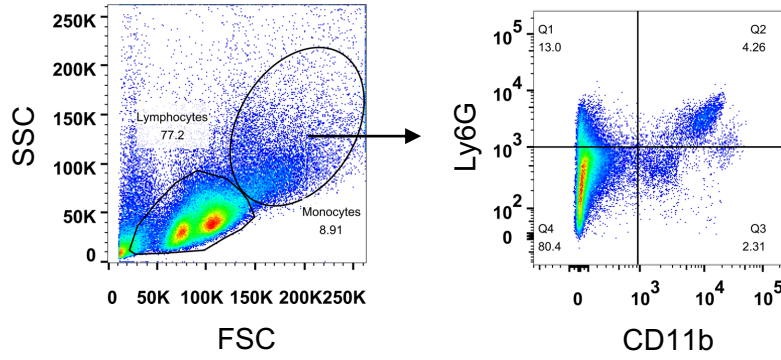

**C**

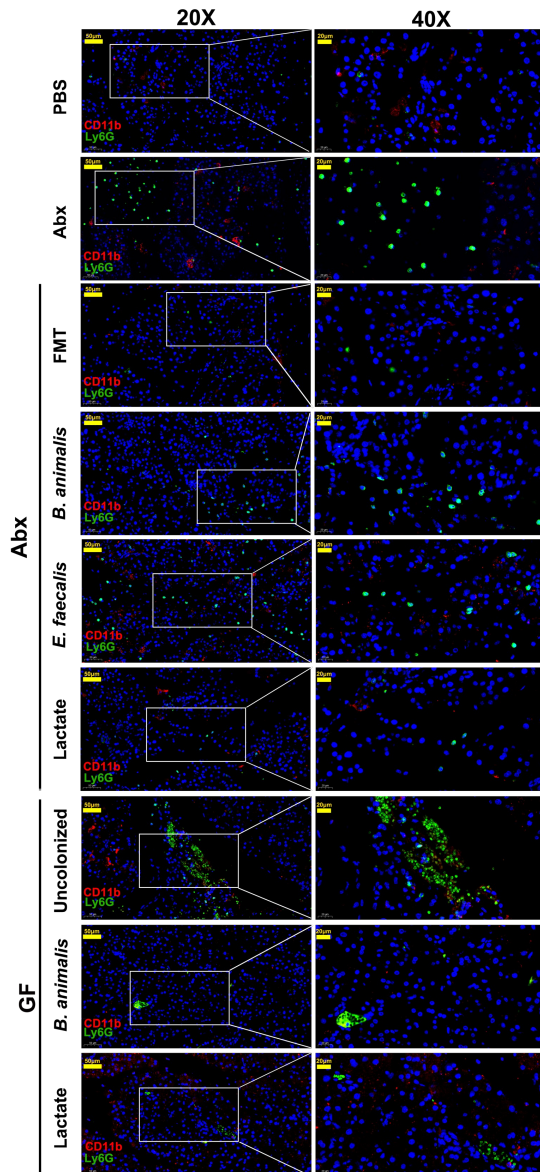

**D**

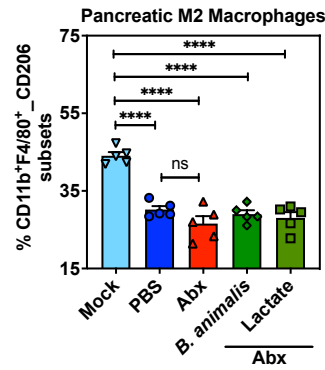

**F**

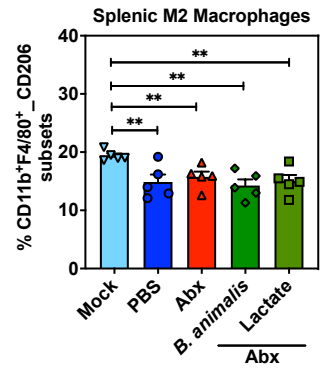

**E**

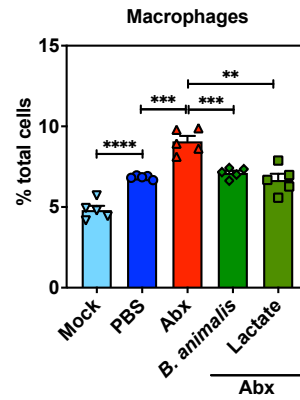

A

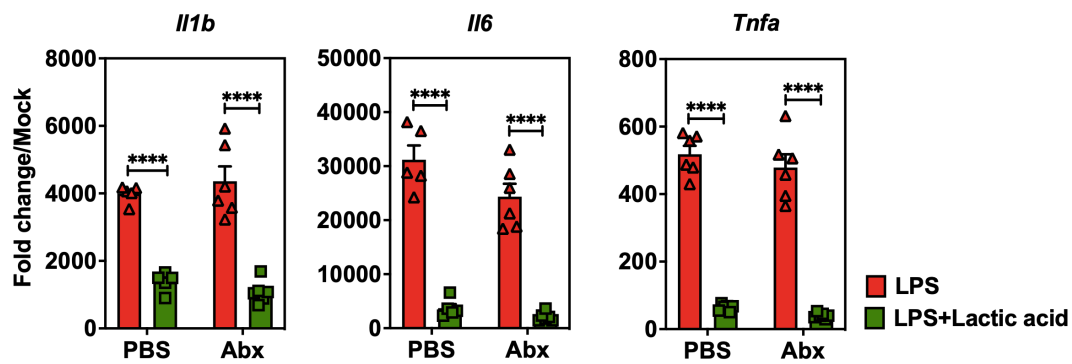

B

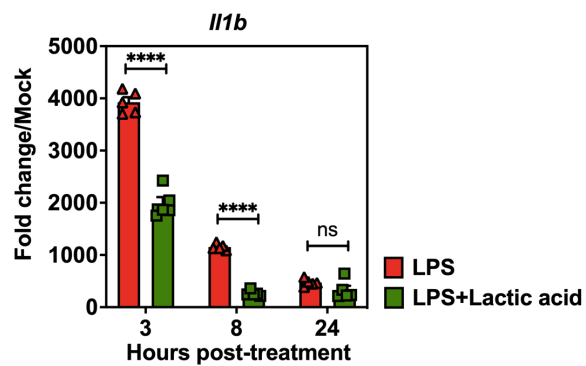

Fig. S5

A

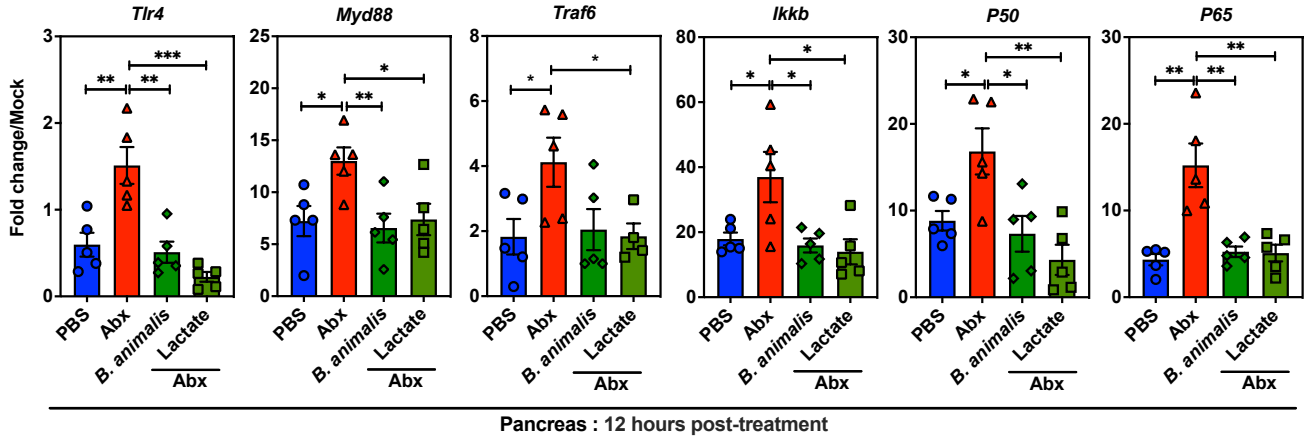

B

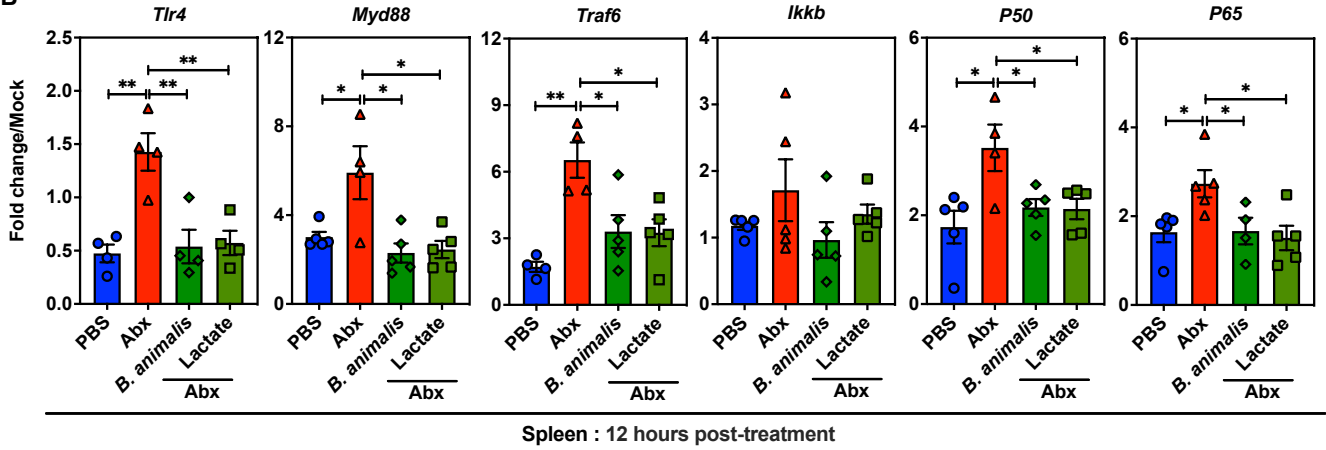

C

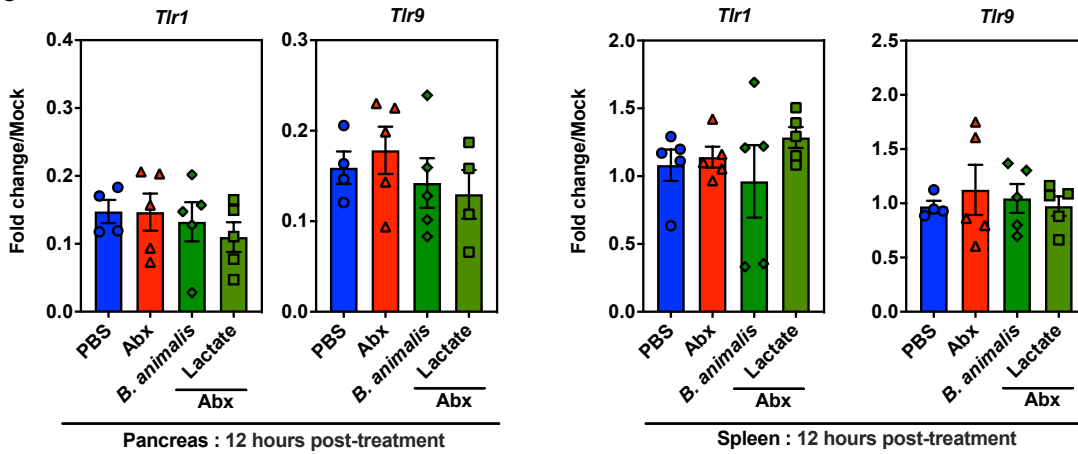

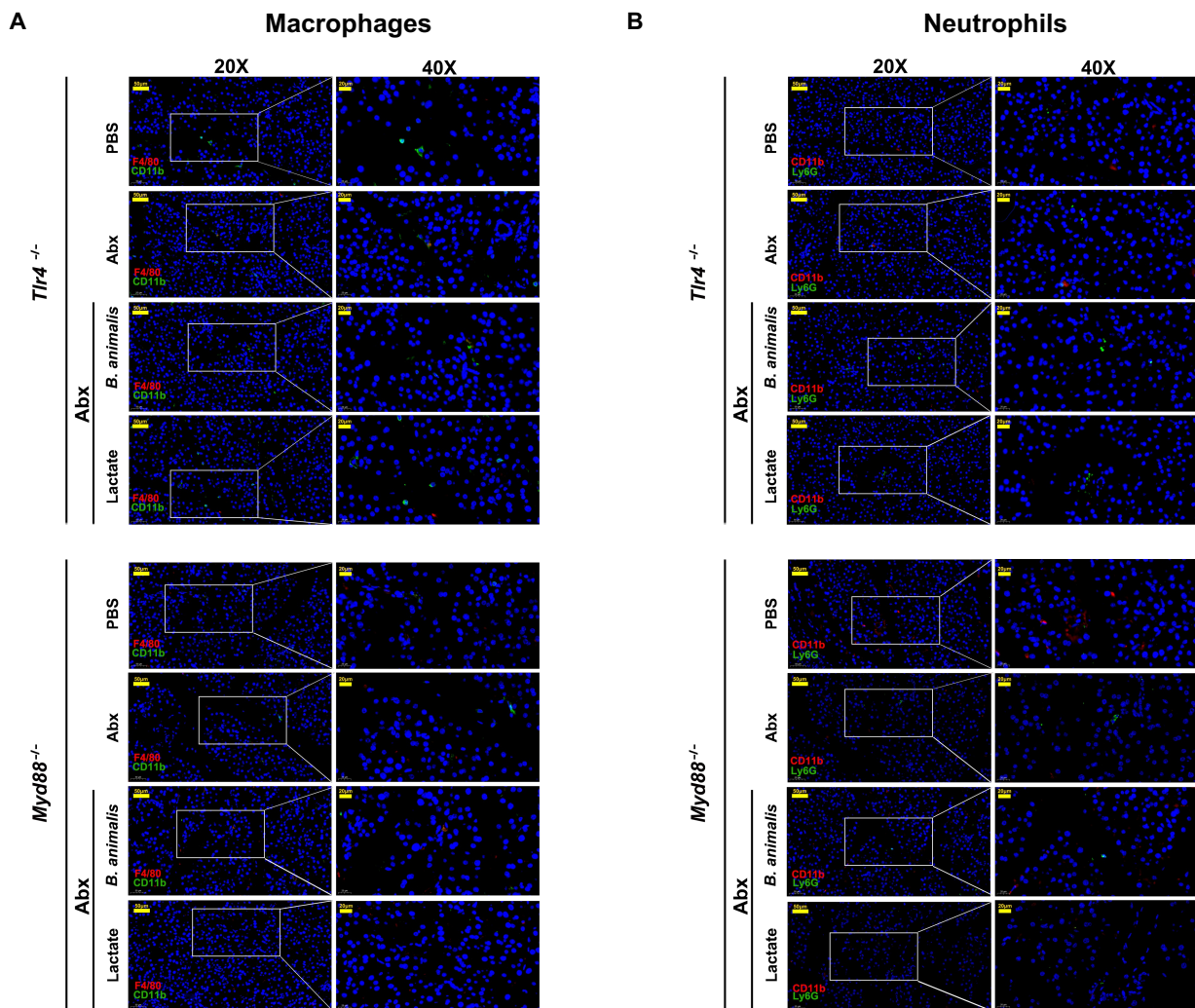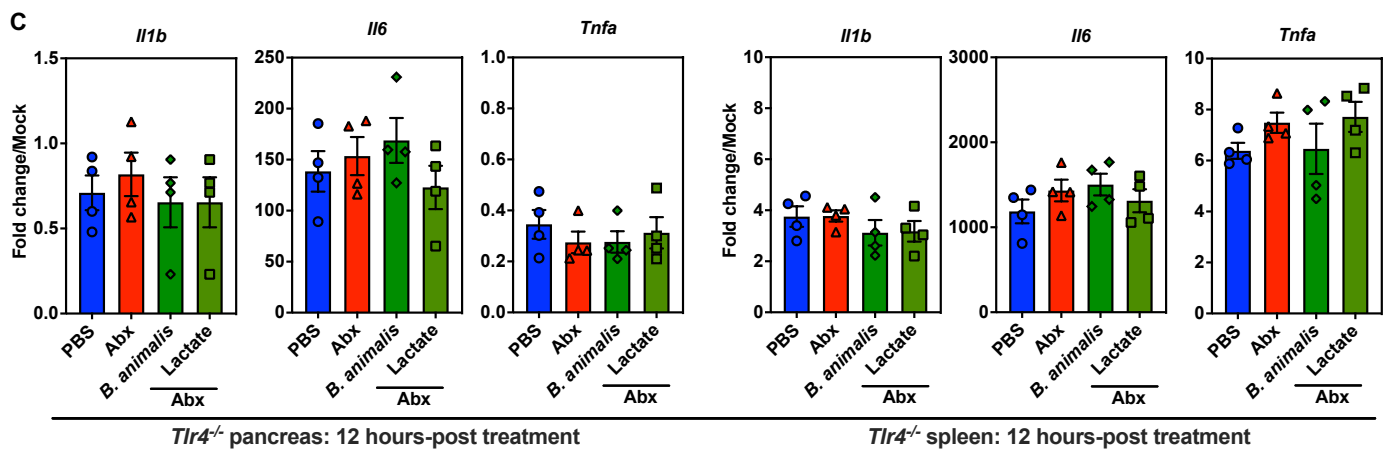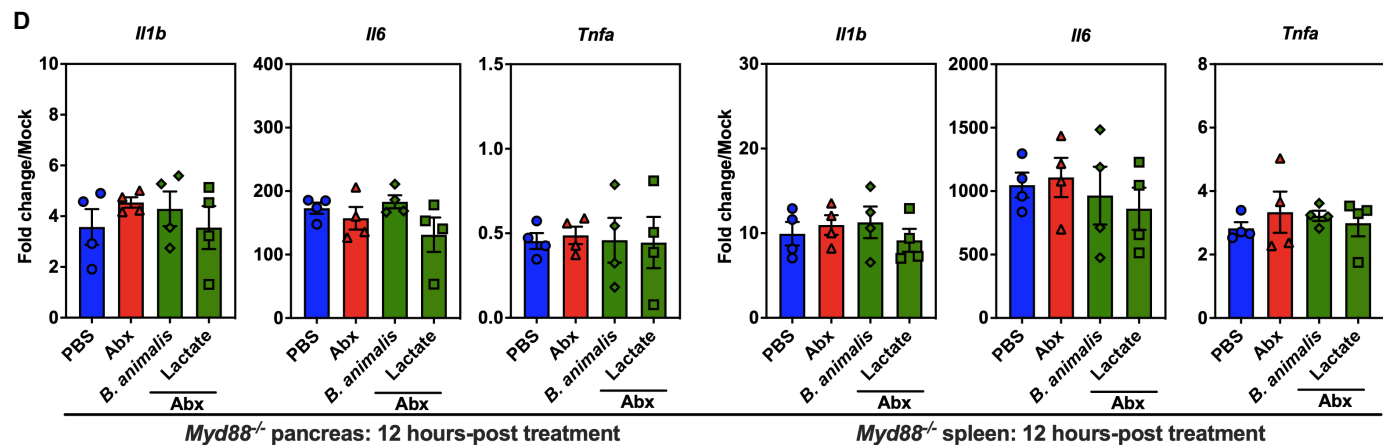

Fig. S7

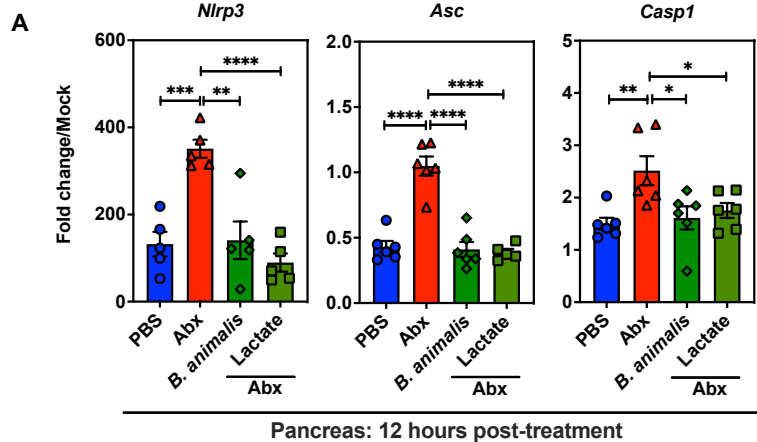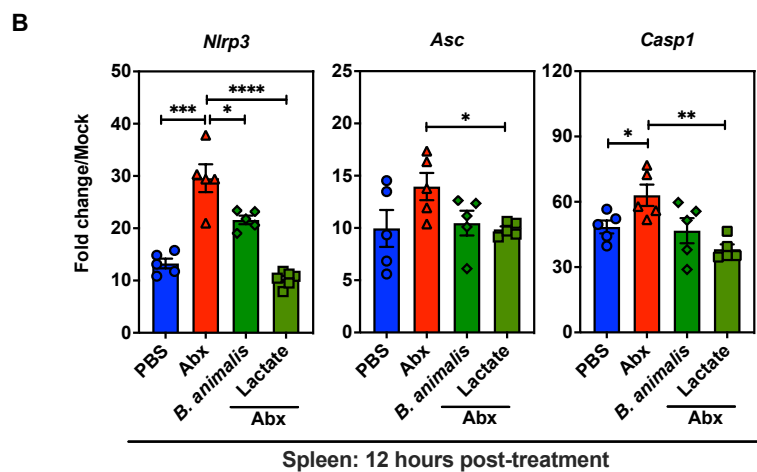

**A** Macrophages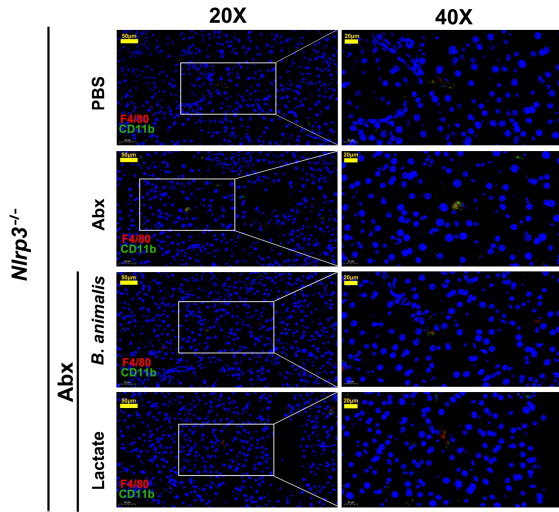**B** Neutrophils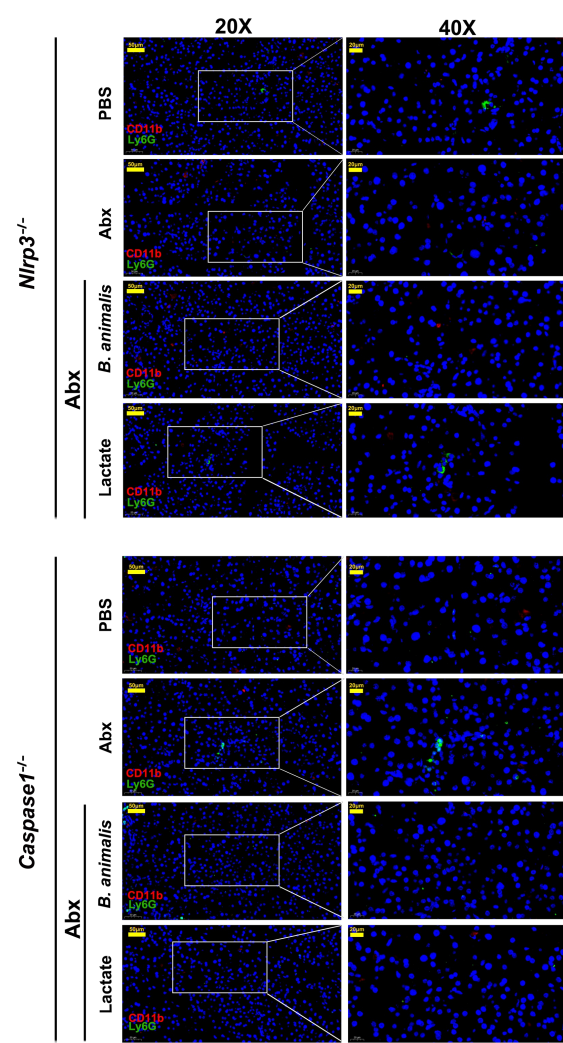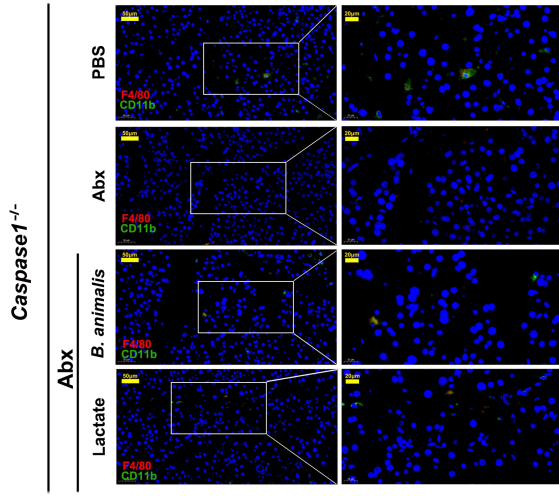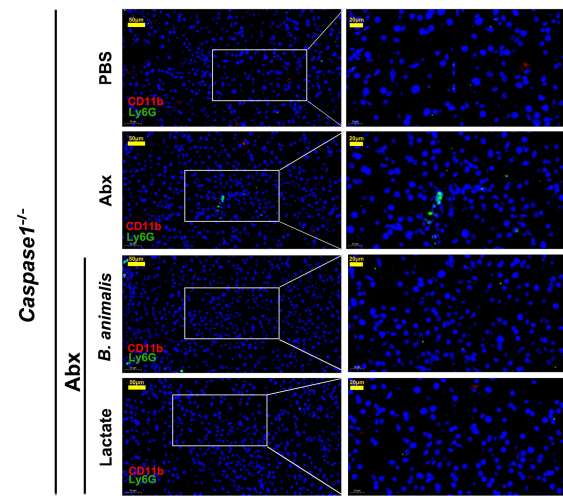**C**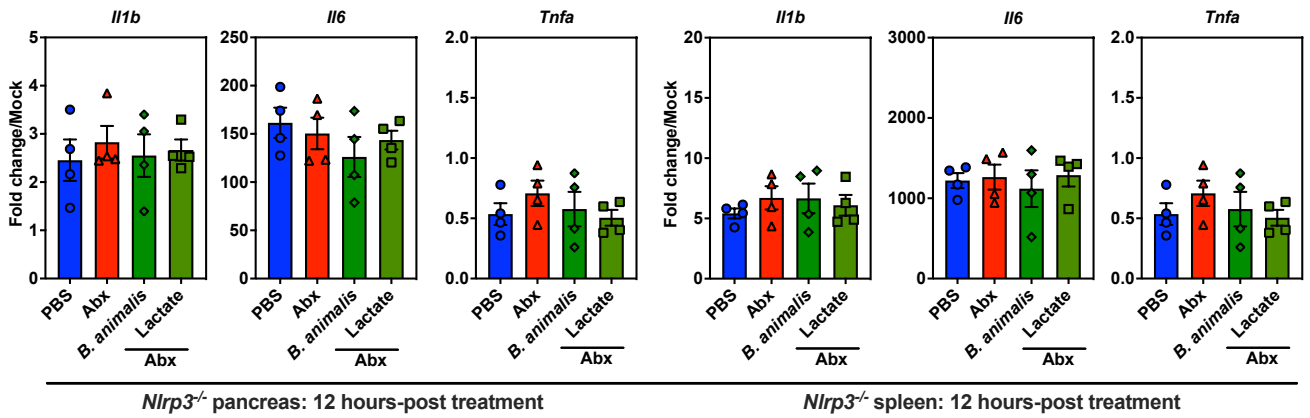**D**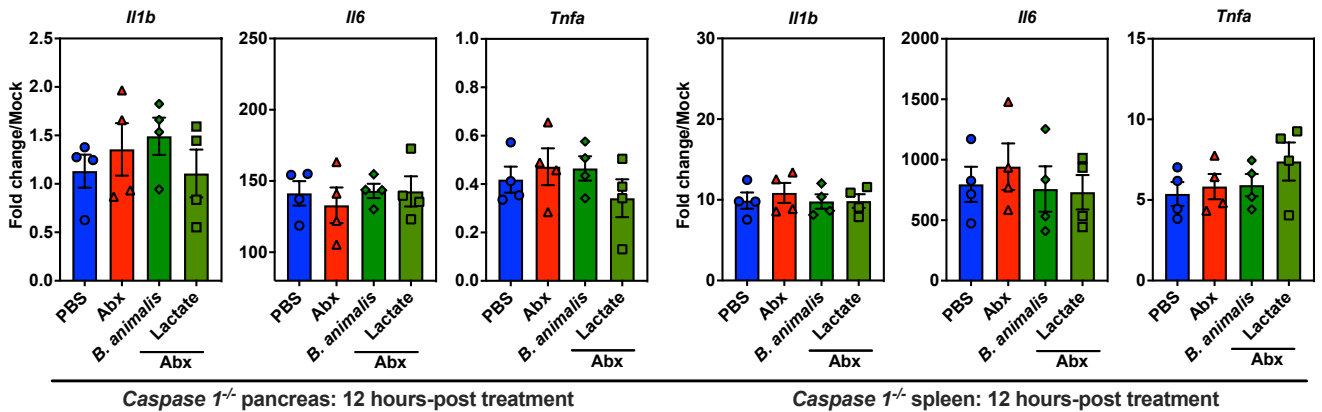

Fig. S9

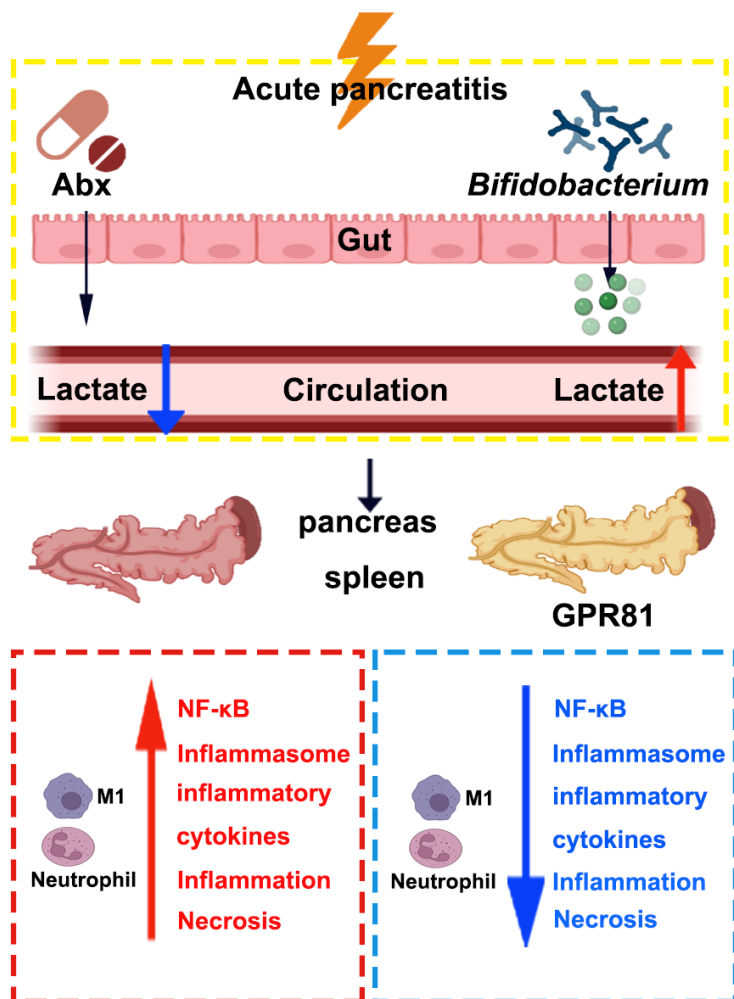

Supplement: Supplemental Material [file KGMI_A_2127456_SM6553.zip › Supplementary figures for submission (1).pdf]
